# Polypept(o)ide Based Biodegradable Cylindrical Polymer Brushes: Controlling Size, Shape, Surface Functionality, and Stability

**DOI:** 10.1021/acsami.5c15018

**Published:** 2025-10-02

**Authors:** Christine Ilona Seidl, Bonan Zhao, Xinye Gao, Rüdiger Berger, Lin Jian, Kaloian Koynov, Meike Gangluff, Rivka Fontijn, Lu Su, Jeroen Bussmann, Heyang Zhang, Matthias Barz

**Affiliations:** † Leiden Academic Center for Drug Research (LACDR), 4496 Leiden University, Einsteinweg 55, Leiden 2333CC, The Netherlands; § Department of Dermatology, University Medical Center of the Johannes Gutenberg University Mainz, Langenbeckstraße 1, Mainz 55131, Germany; ∥ Physics at Interfaces, 28308 Max Planck Institute for Polymer Research, Ackermannweg 10, Mainz 55128, Germany; ⊥ Department Chemie, 9182 Johannes Gutenberg University Mainz, Duesbergweg 10-14, Mainz 55131, Germany; # BioNTech SE, An der Goldgrube 12, Mainz 55131, Germany

**Keywords:** cylindrical polymer brushes, polypept(o)ide, controllable size and shape, thermal stability, surface functionality, “stealth-like” effect

## Abstract

Cylindrical polymer brushes (CPBs) enable remarkable control over nanoparticle properties solely through sequential polymerization. The spatial dimensions and functionality of the resulting polymeric nanoparticles can be adjusted by the ratio of backbone to side chain length and the chemical nature of both parts. In this work, we present a convenient and straightforward synthetic pathway to polypept­(o)­ide-based CPBs using a “grafting-from” strategy utilizing poly-l-lysine (pLys) as the macroinitiator backbone and polysarcosine (pSar) as the side chain. End-capping of pSar chains with azido-butyric acid pentafluorophenyl ester enables facile surface functionalization by click chemistry (e.g., dye labeling). This strategy allows for straightforward control over nanoparticle size (*R*_h_ from 12 to 41 nm), shape (aspect ratio from 1.7 to 8.3), and molecular weights (from 350 to 2980 kg mol^–1^). Despite the high grafting density of pSar side chains from the pLys backbone (>85%), enzymatic degradation is feasible by the natural protease B from*Streptomyces griseus*and enables the analysis of pSar side chains upon cleavage (*Đ* = 1.03–1.04). Interestingly, these CPBs exhibit thermal stability in phosphate-buffered saline at elevated temperatures (60 °C for 24 h) and display notable circulation in zebrafish embryos (up to 3 days). Therefore, CPBs based on polypept­(o)­ides not only allow for precise tuning of size, shape, and surface functionality but also display high biocompatibility and extended circulation time in zebrafish, leveraging the stealth-like properties of pSar.

## Graphical abstract

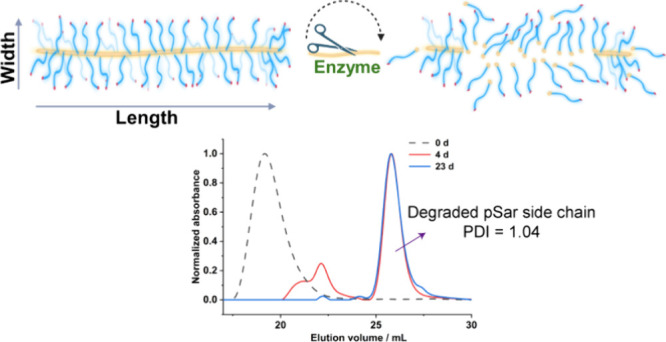

## Introduction

In recent years, research on cylindrical polymer brushs (CPBs) and their applications in biomedicine has increased dramatically.^1–5^ Their distinct architecture, consisting of a linear polymer backbone with one or more polymeric side chains per repeating unit of backbone, offers adaptability to a wide range of applications.^6–8^ The dense grafting of side chains gives rise to strong steric repulsion between themselves, ultimately leading to a stretched backbone and formation of molecular nonspherical nanostructures.^9^ The side chains can be tailored with respect to the microstructure (block or gradient copolymers) and functionality. Taking advantage of this architecture and combining it with stealth-like polymers CPBs become a versatile platform for biomedical use, particularly in drug delivery, gene transfer, and diagnostic approaches.^10–17^

The physicochemical characteristics of CPBs can be tailored by adjusting the parameters, such as the backbone length, the side-chain length, and the grafting density along the backbone. The three main synthetic strategies, “grafting-through”,^18,19^ “grafting-onto”,^20,21^ and “grafting-from”,^22,23^ offer diverse approaches for the synthesis of CPBs. In the “grafting-from” strategy, a backbone polymer carrying a predetermined number of initiating groups is first prepared, and these sites subsequently trigger the growth of side chains. This gradual extension of the side chains helps to minimize steric hindrance, a common limitation encountered in the “grafting-through” and “grafting-onto” methods. While for “grafting-through”, ring-opening metathesis polymerization (ROMP) is widely applied,^18,24^ the “grafting-from” strategies rely on the controlled polymerization from a macroinitiator. The commonly applied approaches for side chain synthesis include controlled radical polymerizations, such as atom transfer radical polymerization (ATRP),^25–27^ reversible addition–fragmentation chain transfer (RAFT),^27,28^ or nitroxide-mediated polymerization (NMP),^29,30^ and the nucleophilic ring-opening polymerization (ROP) of α-amino acid *N*-carboxyanhydrides.^22,25,31^

Notably, the hydrocarbon backbones that are widely used are not degradable, which limits their potential systemic therapeutic applications. To address this issue, researchers have developed peptide-based CPBs to enhance their biodegradability. Rhodesa and Deming reported the first polypeptide-based brush with high grafting density through “the grafting-from” method using a stepwise tandem cobalt and nickel catalysis.^22^ Kramer and co-workers reported grafting-from glycopolypeptide brushes, where the backbone and side chain lengths were precisely altered with high glycan grafting density.^32^ In collaboration with the group of Schmidt, we have earlier described a worm-like CPB with poly­[*N*-(6-aminohexyl)­methacrylamide] (PAHMA)^33^ as the backbone and polysarcosine (pSar, poly­(*N*-methyl glycine))^34^ as side chains, primarily for specific numbers of siRNA formulation or antibody conjugation. The transition from PAHMA to a polypeptide backbone is desirable to obtain CPBs fully based on endogenous amino acids. Additionally, as research has shown, it is favorable to move away from the widely used polyethylene glycol (PEG) for improved effectiveness. A new alternative pSar shows potential in biomedical applications due to its reduced immunogenicity^35,36^ and improved pharmacokinetic profile^37^ compared to PEG. CPBs based on polypept­(o)­ides^38–40^ are directly accessible, which integrate the desirable properties of polypeptoids like pSar and polypeptides as already demonstrated for other polymer architectures.^35,41,42^ Especially, pLys, derived from one of the 20 naturally occurring amino acids, can serve as an initiator for branch points via NCA polymerization by utilizing its ε-amino groups.^43^ pLys itself can be synthesized by controlled living ROP of the corresponding NCA utilizing several techniques, such as transition metal catalysts,^44^ organic acids,^45–49^ bases,^50,51^ and other optimized reaction conditions.^52,53^ Additionally, polypeptoids synthesized via ROP do not rely on an activated monomer mechanism, which allows accurate regulation of chain length and facilitates high grafting densities, making this approach especially suitable for the “grafting-from” synthesis of CPBs.^54,55^

In this study, five well-defined pLys with varying repeat units were used as backbones (*DP*_n_ ≈ 50, 100, 250, 300, and 800) to initiate Sar-NCA polymerization for the synthesis of biomedical meaningful CPBs with pSar side chains. The obtained peptobrushes (PB), namely, p­(L)­Lys_50_-*g*-pSar_40_(N_3_) (**PB50**), p­(L)­Lys_100_-*g*-pSar_25_(N_3_) (**PB100**_**Short**_), p­(L)­Lys_100_-*g*-pSar_40_(N_3_) (**PB100**), p­(L)­Lys_250_-*g*-pSar_40_(N_3_) (**PB250**), p­(L)­Lys_300_-*g*-pSar_45_(N_3_) (**PB300**), and p­(L)­Lys_800_-*g*-pSar_40_(N_3_) (**PB800**), were characterized using size-exclusion chromatography (SEC), proton nuclear magnetic resonance (^1^H NMR), diffusion-ordered spectroscopy (DOSY-NMR), multiangle dynamic laser light scattering (MADLS), multiangle static laser light scattering (MASLS), scanning force microscopy (SFM), circular dichroism (CD) spectroscopy, and fluorescence correlation spectroscopy (FCS). Their biodegradability (with natural proteases*Streptomyces griseus*), thermal stability, and biological performance when exposed to cells and zebrafish embryos were also evaluated.

## Experimental Section

### Materials

All reagents and solvents were obtained from commercial suppliers and used without additional purification, unless noted otherwise. *N*,*N*-Dimethylformamide (DMF, 99.8%, Fisher Scientific) was further dried over molecular sieves and purified by repeated freeze–pump–thaw cycles (final water content <50 ppm). Milli-Q water (18.2 MΩ·cm, TOC < 5 ppm) was prepared with a MILLI-Q Reference A+ system. Hexafluoroisopropanol (HFIP) and potassium trifluoroacetate were provided by Fluorochem. Deuterated solvents were purchased from Deutero GmbH. Poly-l-lysine trifluoroacetate was obtained from Alamanda Polymers, Inc. UltraPure agarose, trypan blue (0.4%), trypsin/EDTA, Dulbecco’s modified Eagle’s medium/F12 (DMEM/F12), and MTT (3-(4,5-dimethylthiazol-2-yl)-(4–2,5-diphenyl tetrazolium bromide) were purchased from Thermo Fisher Scientific (Landsmeer, The Netherlands). RPMI1640, l-glutamine, PEN-STREP (10,000 U mL^–1^, penicillin, 10,000 U mL^–1^ streptomycin), and PBS (without Ca^2+^ or Mg^2+^) were bought from Lonza Bioscience (Verviers, Belgium). Fetal bovine serum was purchased from SERANA (Brandenburg, Germany). Human serum, pooled from six healthy donors, was provided by the transfusion center of the Medical Department of Johannes Gutenberg University Mainz.

### Characterization Methods

Proton nuclear magnetic resonance (^1^H NMR) spectra were collected on a Bruker Avance II 400 spectrometer (400 MHz) at room temperature. DOSY NMR measurements were carried out on the same instrument using the bipolar pulse sequence (stebpgp1s) with parameters d20 = 0.2 and p30 = 2750 μs, applying gradient strengths ranging from 5 to 95%. Solvent peaks were used for spectral calibration, and data were processed with MestReNova 14.0.0 (Mestrelab Research S.L.).

#### Melting Points

The melting points of Lys­(Z)-NCA and Sar-NCA were measured with a METTLER FP62 apparatus (METTLER WAAGEN GmbH). Around 2–3 mg of recrystallized sample was sealed in a capillary tube and heated from room temperature at 1 °C/min under ambient pressure. The onset of melting was taken as the recorded value. Each measurement was repeated twice to confirm reproducibility.

Attenuated total reflectance Fourier transform infrared (ATR-FTIR) spectroscopy was collected on a JASCO FT/IR-4100 spectrometer equipped with a MIRacle ATR accessory (Pike Technologies). Data were processed using Spectra Manager 2.0 (JASCO). The progress of NCA polymerization was followed by FT-IR, and completion was verified by the disappearance of carbonyl absorption bands at 1858 and 1788 cm^–1^.

Size-exclusion chromatography (SEC) analyses were performed on a Jasco system operated at 40 °C with a flow rate of 1.0 mL min^–1^. Two different eluents were applied: HFIP and PBS. For HFIP-SEC, hexafluoroisopropanol containing 3 g L^–1^ potassium trifluoroacetate was used as the mobile phase. Separation was achieved with PFG columns (100 and 1000 Å, 7 μm particle size, modified silica gel) obtained from PSS Polymer Standards Service GmbH. Detection was carried out with a Jasco UV-2075+ UV detector at 230 nm, using toluene as the internal standard, and chromatograms were processed with PSS WinGPC software. For aqueous SEC, PBS (Sigma-Aldrich) was employed as the eluent in combination with a TSKgel GMPWXL 808025 column (Tosoh Corporation), designed for water-soluble polymers. UV detection at 230 nm was again performed with the Jasco UV-2075+, and the raw data were evaluated using Agilent ChemStation 1.0.

Single-angle dynamic light scattering (DLS) measurements and ζ-potential measurements were carried out on a ZetaSizer Nano ZS (Malvern Instruments, Worcestershire, UK) equipped with a He–Ne laser (λ = 632.8 nm). Measurements were performed at 25 °C with backscattering detection at 173°. Samples (0.3 mg mL^–1^ in PBS) were filtered through 0.2 μm Millex-GV membranes before measurement. ζ-potential values were determined in 10 mM HEPES buffer at pH 7.4.

#### Multiangle Dynamic Light Scattering (DLS) and Static Light Scattering (SLS)

Quartz cuvettes (Hellma, Müllheim, Germany) were cleaned with dust-free distilled acetone and stored in a clean flow box prior to use. Solutions were filtered into the cuvettes through Millex-GV membranes (0.2 μm pore size). DLS experiments were carried out at 20 °C with a Uniphase He–Ne laser (λ = 632.8 nm, 22.5 mW) and an ALV/CGS-8F SLS/DLS 5022F goniometer, equipped with eight ALV 7004 correlators and eight ALV/High QEAPD avalanche photodiode detectors. Autocorrelation functions were processed with ALV-Correlator software, and the reported hydrodynamic radius corresponds to the inverse *z*-average, <1/*R*_h_>_*z*_^–1^. SLS measurements were obtained at eight scattering angles between 26° and 122° for concentrations ranging from 100 to 400 μg mL^–1^. Data were analyzed by constructing Zimm plots using ALVStat 4.31 (ALV, Germany), providing *M*_w_, *A*_2_, and the square root of the *z*-averaged radius of gyration (*R*_g_ = <*S* ^2^>_*z*_^1/2^), with a refractive index increment (d*n*/d*c*) of ∼0.176 mL g^–1^ for pSar brushes in PBS or MeOH.^33^

#### Fluorescence Correlation Spectroscopy (FCS)

FCS experiments were conducted on a commercial confocal microscope (LSM 880, Carl Zeiss, Jena, Germany) equipped with a C-Apochromat 40×/1.2 W water-immersion objective. Excitation of the Alexa Fluor 647 was achieved with a fiber-coupled 633 nm He–Ne laser. Emission was collected at 655–699 nm using a Quasar spectral detection unit (Carl Zeiss). For each measurement, 200 μL of solution was transferred into an 8-well polystyrene chambered cover glass (Nunc Lab-Tek, Thermo Fisher Scientific, Waltham, MA). The confocal volume was positioned 30 μm above the glass surface, and 20 runs of 10 s each were performed at 23 °C. Experimental autocorrelation curves were fitted with a standard analytical model:

$$G\left(\tau \right)=1+\left[1+\frac{f_{T}}{1-f_{T}}e^{-\tau /\tau_{T}}\right]\frac{1}{N}\sum_{i=1}^{m}\frac{f_{i}}{\left(1+\frac{\tau }{\tau_{D,i}}\right)\cdot \sqrt{1+\frac{\tau }{S^{2}\cdot \tau_{D,i}}}}$$

where *f*_T_ and τ_T_ denote triplet fraction and relaxation time, respectively, *N* is the average particle number, τ_D,*i*_ is the diffusion time of the *i-*th type of species, *f*_*i*_ is the fraction of component *i* (1 ≤ *i* ≤ *m*), and *S* is the structure parameter, *S* = *z*_0_/*r*_0_, where *z*_0_ and *r*_0_ represent the axial and radial dimensions of the observation volume, respectively. Fitting was carried out with ZEN 3.0 software (Carl Zeiss) using a two-component diffusion model, τ_D1_ was fixed and calibrated by 10 nM Alexa Fluor 647 in PBS. This analysis yielded the fraction of free dye (*f*_1_) and CPBs (*f*_2_), τ_D2_, *N*, and *S*. The diffusion coefficients of species *D*_*i*_ are related to the respective diffusion times τ_D,*i*_ and the radial dimension *r*_0_ of *V*_obs_ by *D*_*i*_ = *r*_0_^2^/(4τ_D,*i*_). By inserting *D*_*i*_ into the Stokes–Einstein equation, the hydrodynamic radius can be calculated as $R_{h}=\frac{k_{B}\cdot T}{6\cdot \pi \cdot \eta \cdot D}$ , here, *k*_B_ is the Boltzmann constant, *T* is the temperature, and η is the viscosity of the solvent. Brightness was calculated as $B=\frac{I}{N}$ , where *I* is the average fluorescence intensity, and the conjugated number of dyes per CPB was calculated by the brightness of CPB divided by the brightness of Alexa Fluor 647. Alexa Fluor 647 was used for the calibration of the confocal volume *V*_obs_.

Scanning force microscopy (SFM) images were acquired in tapping mode with a Dimension ICON instrument (Bruker) using OTESPA probes (nominal resonance frequency 300 kHz, spring constant 26 N m^–1^). Samples were prepared by drop-casting 20 μL of a particle dispersion (1 mg L^–1^ in MQ water) onto freshly cleaved mica, followed by overnight drying under a vacuum (1 kPa) at room temperature. Images were analyzed using Gwyddion, while dimensions were extracted with Nano Measure 1.2. Gaussian distributions of length and width were fitted with Origin software.

Circular dichroism (CD) spectra were recorded on a Jasco J-815 spectrometer at 20 °C and processed with Spectra Manager 1.53.04. Measurements were performed in MQ water (0.1 g L^–1^) by using a 1 mm path-length quartz cuvette. Mean residue ellipticity (θ_MR_) was calculated with *C*_M_ = 0.1 g L^–1^, *l* = 0.1 cm, and molar masses of repeat units.

$$\theta_{\mathrm{MR}}=\frac{\theta \cdot M_{\mathrm{repeating}\;\mathrm{unit}}}{C_{M}\cdot l}\left[\deg\cdot {\mathrm{cm}}^{2}\cdot {\mathrm{mol}}^{-1}\right]$$

#### Biodegradability

Protease from*Streptomyces griseus* (Type XIV, Sigma-Aldrich) was dissolved at 12 mg mL^–1^ in buffer (10 mM sodium acetate, 5 mM calcium acetate, pH 7). Peptobrush samples were dissolved in the same buffer at 8 mg mL^–1^, and equal volumes were mixed to a final polymer concentration of 4 mg mL^–1^. Enzymatic degradation was carried out at 37 °C and 500 rpm in an Eppendorf ThermoMixer C. At defined time points, aliquots were withdrawn, lyophilized, and analyzed by HFIP-SEC relative to polysarcosine standards.^54^ Protease solubility in HFIP was limited, but a reference SEC trace was nevertheless obtained (see Figure S25).

#### Synthesis of Sarcosine *N*-Carboxyanhydride (Sar-NCA)

The preparation of Sar-NCA followed a reported protocol with slight adjustments.^38^ Vacuum-dried sarcosine (21.5 g, 240 mmol, 1.0 equiv) was charged into a flame-dried three-neck round-bottom flask under nitrogen. Absolute THF (300 mL) was added, and diphosgene (23.3 mL, 190 mmol, 0.8 equiv) was introduced dropwise by syringe. The resulting suspension was heated under gentle reflux for 2 h, affording a clear solution. The mixture was subsequently purged with a continuous nitrogen stream for another 2 h with the exit gas passed through aqueous NaOH traps to quench excess phosgene. Removal of the solvent under reduced pressure gave an amorphous residue, which tested free of HCl and phosgene by the AgNO_3_ assay. The crude solid was dissolved in dry THF (40 mL) and precipitated in dry hexane (300 mL). After filtration under nitrogen and drying in a nitrogen stream for 1 h, the product was further purified by sublimation at 80 °C under 10^–3^ mbar for 2 h. The crystalline product (137 mmol, 58% yield, mp = 103 °C) was transferred into a Schlenk tube in a glovebox and stored at −80 °C. ^1^H NMR (400 MHz, CDCl_3_): δ [ppm] = 4.14 (2H, s, −CH_2_–CO−), 3.04 (3H, s, −CH_3_).

#### Synthesis of *N-*ε-Benzyloxycarbonyl-l-lysine-*N*-carboxyanhydride (Lys­(Z)-NCA)

The synthesis of Lys­(Z)-NCA was adapted from a published method with modifications.^56^ Z-protected lysine (20 g, 70 mmol, 1.0 equiv) was placed into a predried three-neck flask fitted with a reflux condenser, dropping funnel, and septum and suspended in dry THF (100 mL). The mixture was heated to 70 °C, and diphosgene (6.6 mL, 56 mmol, 0.8 equiv) was added dropwise over 30 min. Stirring continued at 70 °C until the solids were fully dissolved (30–60 min). To remove residual HCl and phosgene, a stream of dry nitrogen was bubbled through the solution for 2–3 h. The reaction mixture was then concentrated under reduced pressure, and dry hexane was added to induce precipitation. After 1 h at 4 °C, the precipitate was isolated by filtration under nitrogen and washed with hexane. The crude product was redissolved in minimal THF, reprecipitated with hexane, and kept at 4 °C overnight. The solid was again filtered under an inert atmosphere and dried in a nitrogen stream. The resulting Lys­(Z)-NCA (18.5 g, 60.4 mmol, 85% yield, mp = 99.8 °C) was stored in a Schlenk tube at −80 °C. ^1^H NMR (400 MHz, DMSO-*d*_6_) δ [ppm] = 9.14 (1H, s, −CO**NH**–C_α_), 7.40–7.25 (6H, m, **–Ar H**, −**NH**(Z)), 5.00 (2H, s, −COO**CH**_**2**_Ph), 4.37 (1H, t, −**C**_α_ **H**−), 2.97 (2H, q, −**CH**_**2**_NH−), 1.74–1.63 (2H, m, −CH–**CH**_**2**_−), 1.44–1.31 (4H, m, −**CH**_**2**_–**CH**_**2**_−).

#### Synthesis of Azido-Butyric Acid Pentafluorophenyl Ester

γ-Azido butyric acid (2 g, 15.5 mmol, 1.0 equiv) was dissolved in dry THF, and triethylamine (2.15 mL, 31.0 mmol, 2.0 equiv) was added. The solution was stirred at room temperature for 30 min, followed by dropwise addition of pentafluorophenyl trifluoroacetate (5.2 mL, 30.0 mmol, 2.0 equiv) via syringe. The reaction was allowed to proceed overnight at an ambient temperature. Progress was monitored by TLC (silica gel 60 F_254_, *n*-hexane/EtOAc 10:1, *R_f_* = 0.51). After evaporation of THF, the residue was dissolved in dichloromethane and extracted three times with water. The organic layer was dried over MgSO_4_, and the solvent was removed under reduced pressure. The crude product was purified by column chromatography (*n*-hexane/EtOAc 10:1, R_f_ = 0.57) to afford the ester (2.3 g, 51% yield). ^1^H NMR (400 MHz, CDCl_3_): δ [ppm] = 3.46 (2H, *t*, *J* = 6.5 Hz, −CH_2_–CH_2_–**CH**_**2**_–N_3_), 2.80 (2H, t, *J* = 7.2 Hz, −CH_2_–**CH**_**2**_–CH_2_–N_3_), 2.05 (2H, m, −**CH**_**2**_–CH_2_–CH_2_–N_3_).

^19^F NMR (376. MHz, CDCl_3_): δ (ppm) = −152.71 (2F, d, *o*-CF), −157.66 (1F, t, *p*-CF), −162.11 (2F, t, *m*-CF).

#### Synthesis Poly­(*N-*ε-benzyloxycarbonyl-l-lysine)_100_ (p­(L)­Lyz­(Z)_100_)

The synthesis of pLyz­(Z) was adapted from literature and modified.^57^ A stock solution of neopentylamine in dry DCM (1 μL mL^–1^) was prepared, and 1.15 mL of this solution was introduced into a predried Schlenk flask fitted with a stir bar under a nitrogen counterflow. A catalyst solution of 1,3-bis-HFAB (8.2 μmol) in dry DCM (2 mL) was then added, and the mixture was stirred at room temperature for 5 min. Separately, Lys­(Z)-NCA (500 mg, 1.63 mmol) was placed in another predried Schlenk flask, dried under high vacuum, and dissolved in dry DCM to give a 100 mg mL^–1^ stock. An aliquot of this solution (3.0 mL, 300 mg, 0.98 mmol) was transferred into the initiator–catalyst mixture, and polymerization was carried out overnight at room temperature under nitrogen and protected from light. Reaction progress was monitored by FT-IR spectroscopy until the disappearance of the NCA carbonyl absorptions at 1858 and 1788 cm^–1^. Upon completion, the polymer was precipitated into diethyl ether and isolated by centrifugation (5000 rpm, 4 °C, 10 min). The pellet was washed three additional times by redispersion in fresh ether with ultrasonic assistance, followed by centrifugation. The final polymer was obtained after drying under high vacuum (192 mg, 75% yield). ^1^H NMR (400 MHz, DMSO-*d*_6_): δ [ppm] = 8.50–7.70 (br, −**NH**–CO–CH−), 7.50–7.00 (705H, br, −**NH**(Z), −**Ar**–**H**), 5.20–4.70 (204H, br, −COO**CH**_**2**_Ph), 4.35–3.60 (br, −CO–**CH**–NH), 3.25–2.75 (134H, br, −**CH**_**2**_–NH−), 2.50–0.90 (165H, br, −CH–**CH**_**2**_–**CH**_**2**_–**CH**_**2**_–CH_2_–NH_2_), 0.85–0.76 (9H, d, **(CH**_**3**_ **)**_**3**_–C−).

### Synthesis Poly­(*N-*ε-benzyloxycarbonyl-l-lysine)_300_ (p­(L)­Lyz­(Z)_300_)

The synthesis of p­(L)­Lyz­(Z)_300_ was carried out similarly to p­(L)­Lyz­(Z)_100_ (yield: 78%). ^1^H NMR (400 MHz, DMSO-*d*_6_): δ [ppm] = 8.50–7.70 (br, −**NH**–CO–CH−), 7.50–7.00 (1744H, br, −**NH**(Z), −**Ar**–**H**), 5.20–4.70 (602H, br, −COO**CH**_**2**_Ph), 4.35–3.60 (br, −CO–**CH**–NH), 3.25–2.75 (361H, br, −**CH**_**2**_–NH−), 2.50–0.90 (303H, br, −CH–**CH**_**2**_–**CH_2_**–**CH**_**2**_–CH_2_–NH_2_), 0.85–0.76 (9H, d, **(CH**_**3**_ **)**_**3**_–C−).

#### Deprotection of pLys­(Z)

The deprotection of pLyz­(Z) was adapted from literature and modified.^58^ The protected polymer was dissolved in TFA (10 mL) in a 100 mL round-bottom flask and stirred for 1 h until complete dissolution. To this solution, HBr in acetic acid (30%, 1.6 mL, 8.3 mmol) was added while cooling in an ice bath, and the mixture was stirred for an additional 5 h. Excess acid was neutralized by dropwise addition of triethylamine under continued cooling, which resulted in the precipitation of salts. The suspension was then diluted with MQ water (20 mL) and extracted repeatedly with diethyl ether to remove organic impurities and residual acid until the ether layer became neutral. The aqueous fraction was transferred into a preswollen dialysis membrane (MWCO 3.5 kDa) and dialyzed first against 0.05% (v/v) TFA solution (2.0 L, pH 2) in an ice bath for 48 h, followed by dialysis against distilled water (2.0 L) at room temperature for 24 h. Dialysates were exchanged every 2 h during the first 8 h and every 8 h thereafter. The retained solution inside the membrane was collected, and the tubing was rinsed several times to recover any remaining material. The combined solution was finally lyophilized to yield pLys as its TFA salt (yield >50%).

#### Peptobrush Synthesis

Brush polymers were synthesized from poly-l-lysine macroinitiators; the procedure for p­(L)­Lys_100_-*g*-pSar_41_(N_3_) (PB100) is outlined as a representative example (see Table S1).

### Synthesis of Poly-l-lysine_100_-*graft*-polysarcosine_41_(N_3_) (p­(L)­Lys_100_-*g*-pSar_41_(N_3_), PB100)

The poly-l-lysine trifluoroacetate macroinitiator (*DP*_n_ ≈ 100, *M*_w_ = 24,000 g mol^–1^, 8.4 mg, 0.35 μmol, 1.0 equiv) was introduced into a predried Schlenk tube and subjected to azeotropic drying with toluene under vacuum overnight. The resulting solid was dissolved in freshly degassed, dry DMF (1.0 mL) and cooled to 10 °C under nitrogen. DIPEA (7.3 μL, 42 μmol, 1.2 equiv relative to amines) was added to neutralize the TFA counterions, and the solution was stirred for 30 min. Sar-NCA (161.1 mg, 1.4 mmol, 4000 equiv) was dissolved in dry DMF and introduced by syringe. Polymerization was carried out at an overall concentration of β = 100 g L^–1^ and monitored by IR spectroscopy. After 5 days, complete monomer conversion was confirmed, and azido-butyric acid pentafluorophenyl ester (12.4 mg, 42 μmol, 1.2 equiv) was added in DMF. The reaction was continued for 2 days at room temperature.

The resulting azido-functionalized peptobrush was purified by dispersion in MQ water, followed by repeated washing with Amicon Ultra centrifugal filters (MWCO 50 kDa; 4000 rpm, 10 × 10 min). After lyophilization, the product was obtained as a colorless solid (yield: 72.6 mg 73%). ^1^H NMR (400 MHz, D_2_O): δ [ppm] = 4.6–4.0 (85H, br, −CO–**CH**–NH– and −NCH_3_–**CH**_**2**_–CO), 3.4–3.2 (2H, m, −CH_2_–CH_2_–**CH**_**2**_–N_3_), 3.2–2.6 (127H, br, −**CH**_**2**_–NH–CO–, −N**CH**_**3**_–CH_2_–CO−), 2.6–2.2 (br, −CH_2_–**CH**_**2**_–CH_2_–N_3_), 1.8–1.7 (br, −**CH**_**2**_–CH_2_–CH_2_–N_3_), 1.7–1.2 (br, −CH–**CH**_**2**_–**CH**_**2**_–**CH**_**2**_–CH_2_–NH−).

### Poly-l-lysine_50_-*graft*-polysarcosine_40_(N_3_) (p­(L)­Lys_50_-*g*-pSar_40_(N_3_), PB50, Yield: 80%)

^1^H NMR (400 MHz, D_2_O): δ [ppm] = 4.6–4.0 (82H, br, −CO–**CH**–NH– and −NCH_3_–**CH**_**2**_–CO), 3.4–3.2 (2H, m, −CH_2_–CH_2_–**CH**_**2**_–N_3_), 3.2–2.6 (123H, br, −**CH**_**2**_–NH–CO–, – N**CH**_**3**_–CH_2_–CO−), 2.6–2.2 (br, −CH_2_–**CH**_**2**_–CH_2_–N_3_), 1.8–1.7 (br, −**CH**_**2**_–CH_2_–CH_2_–N_3_), 1.7–1.2 (br, −CH–**CH**_**2**_–**CH**_**2**_–**CH**_**2**_–CH_2_–NH−).

### Poly-l-lysine_250_-*graft*-polysarcosine_40_(N_3_) (p­(L)­Lys_250_-*g*-pSar_40_(N_3_), PB250, Yield: 78%)

^1^H NMR (400 MHz, D_2_O): δ [ppm] = 4.6–4.0 (83H, br, −CO–**CH**–NH– and −NCH_3_–**CH**_**2**_–CO), 3.4–3.2 (2H, m, −CH_2_–CH_2_–**CH**_**2**_–N_3_), 3.2–2.6 (127H, br, −**CH**_**2**_–NH–CO–, −N**CH**_**3**_–CH_2_–CO−), 2.6–2.2 (br, −CH_2_–**CH**_**2**_–CH_2_–N_3_), 1.8–1.7 (br, −**CH**_**2**_–CH_2_–CH_2_–N_3_), 1.7–1.2 (br, −CH–**CH**_**2**_–**CH**_**2**_–**CH**_**2**_–CH_2_–NH−).

### Poly-l-lysine_800_-*graft*-polysarcosine_41_(N_3_) (p­(L)­Lys_800_-*g*-pSar_41_(N_3_), PB800, Yield: 75%)

^1^H NMR (400 MHz, D_2_O): δ [ppm] = 4.6–4.0 (84 H, br, −CO–**CH**–NH– and −NCH_3_–**CH**_**2**_–CO), 3.4–3.2 (2 H, m, −CH_2_–CH_2_–**CH**_**2**_–N_3_), 3.2–2.6 (128 H, br, −**CH**_**2**_–NH–CO–, −N**CH**_**3**_–CH_2_–CO−), 2.6–2.2 (br, −CH_2_–**CH**_**2**_–CH_2_–N_3_), 1.8–1.7 (br, −**CH**_**2**_–CH_2_–CH_2_–N_3_), 1.7–1.2 (br, −CH–**CH**_**2**_–**CH**_**2**_–**CH**_**2**_–CH_2_–NH−).

### Fluorescent Labeling

The conjugation of Alexa Fluor 647-DBCO was performed via Strain-promoted Alkine-Azide Conjugation (SPAAC). The procedure was identical for each brush polymer and executed as previously described in literature.^9^ The dye quantities on each brush were determined by FCS (see method section Fluorescence Correlation Spectroscopy (FCS)) to ∼5 dyes per PB100, ∼10 dyes per PB250, and ∼20 per PB800, and <6% of free dye was detected for all polymer solutions.

### Cell Culture

HeLa cells and Raw264.7 cells were maintained in DMEM/F12 containing 10% fetal bovine serum (FBS), 2 mM l-glutamine, and 100 μg mL^–1^ penicillin/streptomycin under standard conditions (37 °C, 5% CO_2_). HeLa cells were detached from the flask by 0.25% trypsin/EDTA. Raw264.7 cells were detached from the bottom of the flask by a cell scraper. Jurkat cells were cultured in RPMI1640 medium supplemented with 10% FBS, 2 mM l-glutamine, and 100 μg mL^–1^ penicillin/streptomycin at 37 °C and 5.0% CO_2_. HeLa cells and Raw264.7 cells were subcultured when the confluency reached 80–90%. Jurkat cell culture medium was renewed every 3 days.

### Biocompatibility

HeLa and RAW264.7 cells were seeded in flat-bottom 96-well plates (Greiner Bio-One, Alphen aan den Rijn, The Netherlands) at 10,000 cells per well, while Jurkat cells were seeded in U-bottom 96-well plates at 40,000 cells per well. All cultures were maintained for 24 h at 37 °C in a humidified incubator with 5% CO_2_ prior to treatment. Cells were washed twice with DPBS(−) and then exposed to PB100, PB250, or PB800 at final concentrations of 0.5, 5.0, or 10 μg mL^–1^. Control wells received DPBS(−) only. After 24 h of incubation, the medium was replaced with 200 μL of MTT solution (0.5 mg mL^–1^). Plates were covered with aluminum foil and incubated for 3 h at 37 °C and 5% CO_2_. Subsequently, the MTT solution was removed and replaced with 100 μL of DMSO. After gentle shaking for 15–20 min, absorbance was recorded at λ_m__a__*x*_ = 590 nm with a reference at 690 nm using a Spark microplate reader (Tecan Austria GmbH). Background-subtracted absorbance values were normalized to those of DPBS(−)-treated controls to calculate relative cell viability.

### Cellular Association

To assess peptobrush uptake, HeLa and RAW264.7 cells were seeded at 10,000 cells per well in flat-bottom 96-well plates, while Jurkat cells were seeded at 40,000 cells per well in U-bottom 96-well plates (all from Greiner Bio-One). Cells were cultured at 37 °C and 5% CO_2_ for 24 h before exposure. Prior to cell treatment, peptobrushes were incubated for 1 h at 37 °C in either PBS or heat-inactivated FBS. They were then applied to cells at a final concentration of 5 μg mL^–1^. After 4 h of incubation, cells were washed twice with PBS, collected, and analyzed by flow cytometry on a CytoFLEX S instrument (Beckman Coulter, Woerden, The Netherlands). Data were processed using FlowJo v10.8.1.

### *In Vivo* Circulation in Zebrafish Embryo

Zebrafish (*Danio rerio*) were maintained and handled according to the guidelines from the Zebrafish Model Organism Database (http://zfin.org) and in compliance with the directives of the local animal welfare committee of Leiden University. Fertilization was performed by natural spawning at the beginning of the light period. Eggs were raised in an incubator at 28.5 °C in egg water (60 μg mL^–1^ Instant Ocean Sea salts). Needles for Duct of Cuvier injection were prepared using a micropipet puller (P-97, Sutter Instruments). A Femtojet (Eppendorf) pump was used for microinjection. The Tg­(kdrl: GFP)^s843^ zebrafish line with GFP expression in endothelial cells was utilized to quantify the circulation time. 2.5 day post fertilization (dpf) zebrafish embryos embedded in 0.4% agarose gel containing 0.01% tricaine were utilized for injection. 1 nL of Alexa Fluor 647-labeled peptobrushes was injected into the Duct of Cuvier of zebrafish embryos, at a concentration of 2 mg mL^–1^ (PB100), 5 mg mL^–1^ (PB250), and 8 mg mL^–1^ (PB800), to keep the total number of peptobrushes and overall fluorescence comparable. After injection, embryos were removed from the agarose and fixed in confocal dishes using 0.4% agarose gel. Confocal z-stacks were captured on a Leica TCS SP8 confocal microscope. For the circulation time test, confocal z-stacks were captured by using a 10× air objective (HCX PL FLUOTAR). Laser intensity, gain, and offset settings for the stacks and sessions were identical. Images were processed using Fiji ImageJ software. The circulation time of these microinjected peptobrushes was quantified using the formula given below.

$$\mathrm{circulation}\;\mathrm{time}\left(\%\right)=\frac{\frac{\mathrm{cadual}\;\mathrm{artery}\;\mathrm{fluorescence}\left(x\;h\right)}{\mathrm{cadual}\;\mathrm{artery}\;\mathrm{fluorescence}\left(1\;h\right)}-\mathrm{background}}{\frac{\mathrm{whole}\;\mathrm{fish}\;\mathrm{fluorescence}\left(x\;h\right)}{\mathrm{whole}\;\mathrm{fish}\;\mathrm{fluorescence}\left(1\;h\right)}-\mathrm{background}}$$

### Statistical Analysis

The results are presented as mean ± standard deviation. Experiments were carried out in at least three replicates on independent days. The significance was determined using one-way or two-way ANOVA with GraphPad Prism 8. The following asterisks indicate statistical significance: *p < 0.05; **p < 0.01; ***p < 0.001, ****p < 0.0001.

## Results and Discussion

Previous research from our group has shown that CPBs with a pSar side chain length of DP 25 or 40 displayed the absence of protein-corona formation and stealth-like properties in mice. As both aspects are non-negotiable for systems in biomedical application, these DPs were also chosen for the peptobrushes used here.^10,37^ We employed commercially available pLys (DP_n_ ≈ 50, 100, 250, and 800, Figure S2B) and in house-synthesized pLys (DP_n_ ≈ 100, 300, 430, and 900, Figures S2A, S7, S9–S10, S14, and S27). All pLys polymers formed random coil secondary structures in an aqueous solution (Figure S3) and can be utilized to initiate Sar-NCA ROP to yield the desired pSar side chains. ROP of Sar NCA (Figure S6) was carried out using purified monomers at 10 °C in dry DMF (<60 ppm water). Completion of polymerization was confirmed by FT-IR spectroscopy through the disappearance of the anhydride carbonyl stretching bands at 1788 and 1858 cm^–1^ (Figure S4). Following full conversion, the terminal groups of the polysarcosine side chains were functionalized with azido-butyric acid pentafluorophenyl ester (Figure S8). This functionalization strategy enables subsequent conjugation to alkyne groups via CuAAC or to DBCO motifs via SPAAC for labeling or bioactive compound attachment^59^ (Scheme 1).

**Scheme 1:**
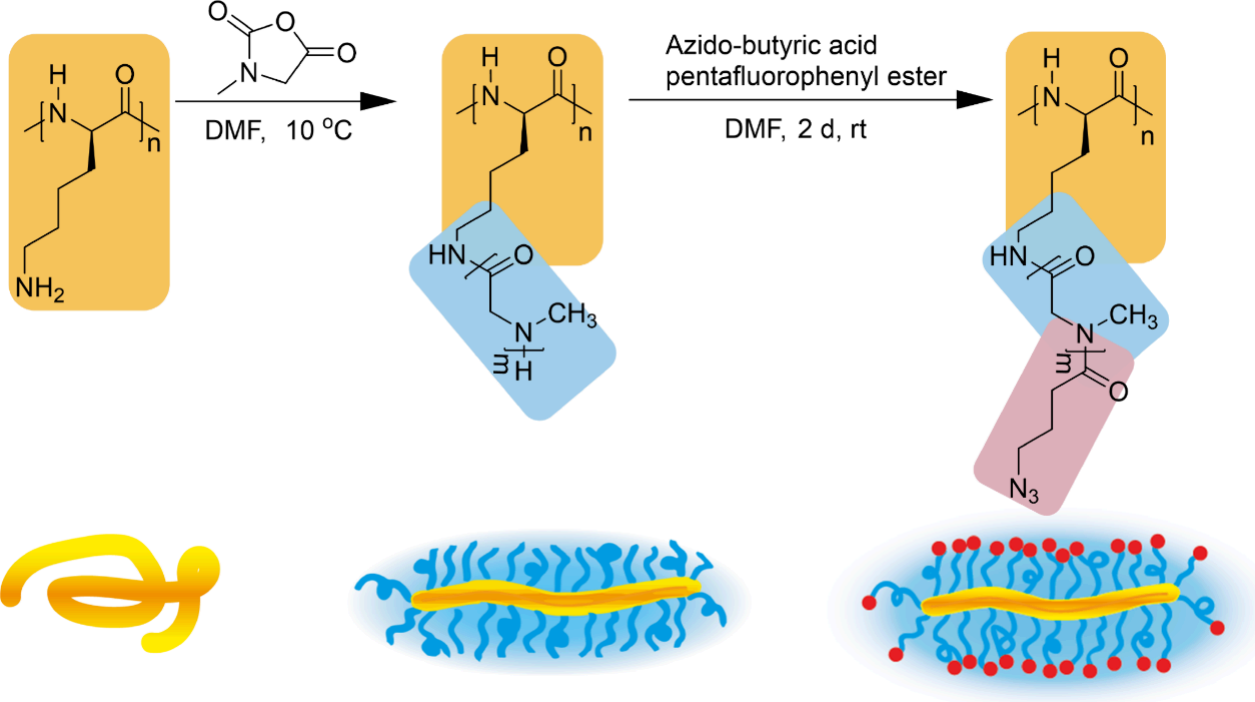
Synthetic Route of the Polypept­(o)­ide Based Cylindrical Polymer Brush

SEC analysis performed in hexafluoroisopropanol (HFIP) revealed monomodal and symmetric molecular weight distributions for all peptobrushes, with narrow dispersities (*Đ* = 1.1–1.3) (as depicted in Figure 1A, Figure S2C, and Table 1). For clarity, we display SEC plots from small (**PB100**) to large brushes (**PB800**) in Figure 1, while all data is present in Table 1. Only **PB800** displayed a low molecular weight tailing in HFIP. Consistent with these findings, aqueous SEC in phosphate-buffered saline (PBS) showed the expected elution volume shifts for **PB100**, **PB250**, and **PB800**, confirming well-controlled polymerization across varying backbone lengths (Figure 1B and Figure S2B). The uniformity of the synthesized polymer brushes was further corroborated by a single CPB species observed in ^1^H-DOSY NMR (Figure 1D, Figures S15–17).

**Figure 1:**
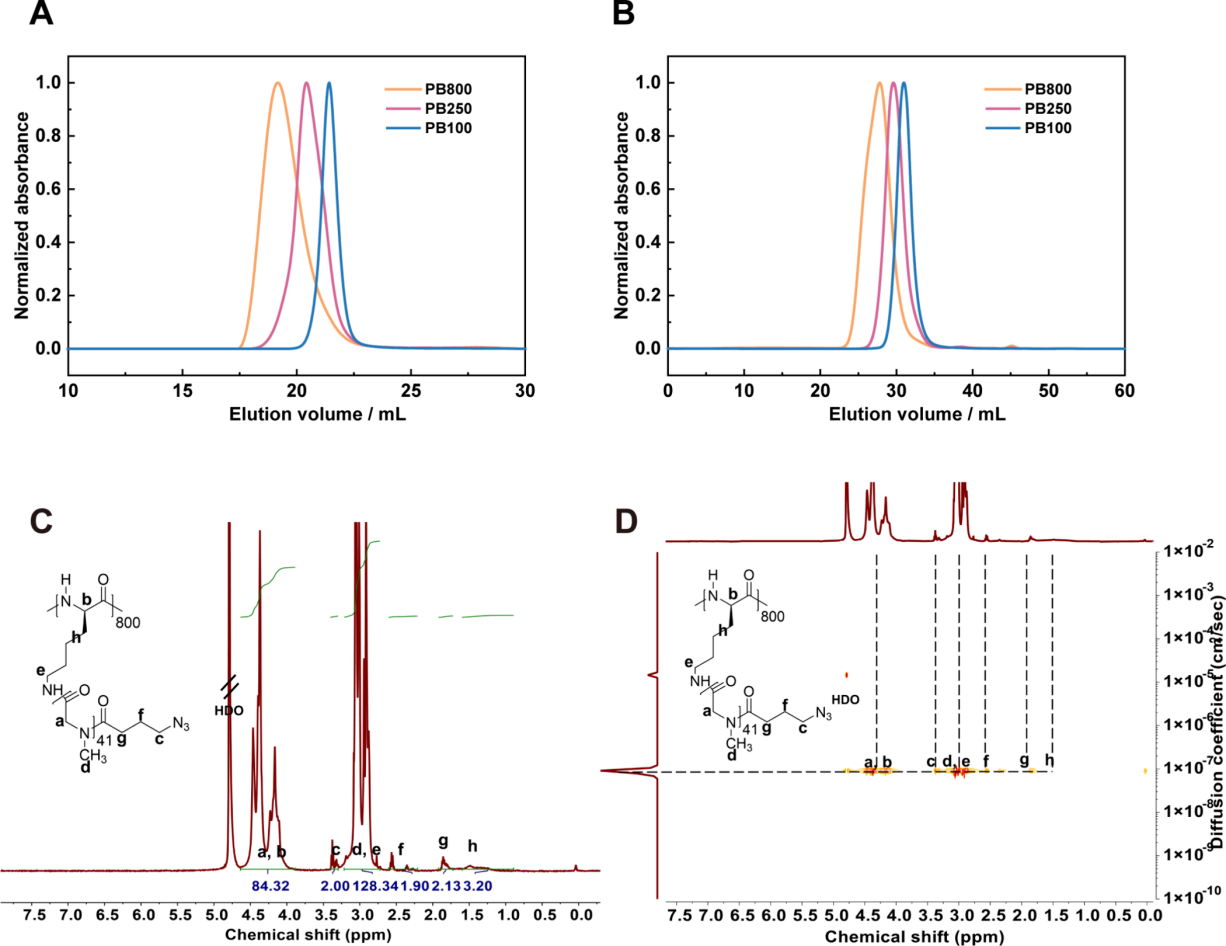
Analytical size-exclusion chromatography in HFIP (A) and PBS (B). Representative ^1^H NMR (C) and DOSY NMR (D) analysis of p­(L)­Lys_800_-*g*-pSar_41_(N_3_) in deuterium oxide (D_2_O).

**Table 1:** Characterization of the Polymer Brush

polymer	*X_n_* (pSar)^a^	*X_n_* (pSar)^b^	*X*_*n*_ (degraded pSar)^c^	*Đ* (side chain, SEC)^c^	*M*_w_ (kg mol^–1^)^b^	*M*_n_ (kg mol^–1^)^a^	grafting density (%)^d^	*Đ* (brush, SEC)^e^	*R*_h_ (mean ± SD, nm)^f^	*PDI* ^f^	ζ-potential (mV)^g^	*R*_h_ (nm)^h^	*A*_2_ (mol L/g^2^)^b^	*R*_*g*_ (nm)^b^	*R*_*h*_ in PBS (nm)^i^	*R*_*h*_ in human serum (nm)^i^	Aspectratio^j^
PB50, p(L)Lys_50_-*g*-pSar_40_(N_3_)	40	41			160	154	96	1.07	8.9 ± 0.1	0.13	+0.6	10	1.0 × 10^–6^	10	-	-	
PB100_Short_, p(L)Lys_100_-*g*-pSar_25_(N_3_)	24	28			220	194	93	1.21	10.1 ± 0.5	0.10	–2.4	11	1.1 × 10^–6^	10	-	-	-
PB100, p(L)Lys_100_-*g*-pSar_40_(N_3_)	41	45	44	1.03	350	320	95	1.15	11.1 ± 0.1	0.09	–1.6	12	1.3 × 10^–6^	12	13	14	1.7
PB250, p(L)Lys_250_-*g*-pSar_40_(N_3_)	40	41	54	1.03	810	790	89	1.32	17.6 ± 0.3	0.12	1.4	19	4.2 × 10^–8^	20	19	21	3.3
PB300, p(L)Lys_300_-*g*-pSar_45_(N_3_)	45	46			1,050	1,030	91	1.28	23.1 ± 0.2	0.14	2.1	22	2.4 × 10^–8^	25	-	-	-
PB430, p(L)Lys_430_-*g*-pSar_76_(N_3_)	76					2400		1.21	31.5 ± 0.3	0.16	–6.3				-	-	-
PB800, p(L)Lys_800_-*g*-pSar_40_(N_3_)	41	49	35	1.04	2,980	2,520	85	1.34	33.0 ± 1.5	0.24	0.7	41	1.5 × 10^–8^	58	40	39	8.3
PB900, p(L)Lys_896_-*g*-pSar_79_(N_3_)	79					5240		1.37	38.1 ± 2.2	0.27	–3.3				-	-	-

a Determined by ^1^H NMR.

b Determined by SLS.

c Determined by HFIP-SEC relative to pSar standards.

d $Grafting\;density=\frac{Mw\left(Brush\right)}{Mw{\left(pSar\right)}^{*}Xn\left(pLys\right)+Mw\left(pLys\right)}$

e Determined by HFIP-SEC relative to PMMA standards.

f Determined by single-angle DLS at 173°.

g Determined in 10 mM HEPES buffer.

h Determined by multiangle DLS at 26°, 58°, 90°, and 122°.

i Determined by FCS.

j Analysis from scanning force microscopy measurements, PB aspect ratio = length/width.

The pSar side chain length was investigated by end-group analysis *via* NMR spectroscopy, indicating peptobrushes with controlled side chain lengths of 41 to 45 (DP_expected_ 40) and 24 (DP_expected_ 25) in the case of short side chains (Table 1, Figure 1C, and Figures S11–S13). In detail, the integration of the methylene resonance of the azide group (3.2–3.4 ppm, m, 2H) was compared with the polysarcosine signal (−CH_2_–CH_2_–**CH**_**2**_–N_3_) in the ^1^H NMR spectra of individual polymers as being confirmed by FT-IR spectrum (Figure S5).

Subsequently, SLS experiments were performed to determine the weight-average molecular weight (*M*_w_) of the peptobrushes (Table 1). The specific refractive index increment was measured by differential refractometry, giving a d*n*/d*c* value of 0.176 cm^3^ g^–1^ in PBS. The obtained *M*_w_ values were 160 kg mol^–1^ for PB50, 220 kg mol^–1^ for **PB100**_**Short**_, 350 kg mol^–1^ for **PB100**, 810 kg mol^–1^ for **PB250**, 1050 kg mol^–1^ for **PB300**, and 2980 kg mol^–1^ for **PB800**. Interestingly, these results closely matched the theoretical molecular weights calculated from DP_n_ of both backbone and side chains. Vice versa, the DP values for pSar side chains calculated from SLS are in accordance with the ^1^H NMR data (Table 1). In addition, the radius of gyration (*R*_*g*_) was determined to be 10 nm for **PB50** and **PB100**_**Short**_ and 12 nm for **PB100**. For the larger PBs, the *R*_*g*_ of **PB250** was measured as 20 nm, while that of **PB300** increased to 25 nm. Notably, the largest **PB800** exhibited an *R*_*g*_ up to 58 nm (Figure S21).

To characterize the hydrodynamic radius (*R*_h_), we employed different light scattering methods (Figure 2 and Figure S18). Single-angle DLS at 173° revealed hydrodynamic radii (*R*_h_) between 9 and 33 nm for **PB50**, **PB100**, **PB250**, and **PB800**, with polydispersity indices (PDIs) ranging from 0.09 to 0.24 (Table 1). Moreover, all polymer brushes displayed nearly neutral ζ-potentials (−1.6 to +1.4 mV), consistent with efficient end-group capping by azide functionalities on the terminal sarcosine residues (Table 1, Figure S18). To generate a higher resolution particle size distribution, peptobrushes were measured by DLS at multiple angles of 26°, 58°, 90°, and 122° (Figure 2A–C, Figures S19–S20). Multiangle DLS analysis autocorrelation function (ACF) showed the absence of aggregates in the samples or during measurements at all angles (Figure S19), while the average *R*_h_ was derived to be 10 nm (PB50), 12 nm (PB100), 19 nm (PB250), and 41 nm (PB800) from the analysis of the autocorrelation functions using CONTIN at four scattering angles. Representative PB50, PB100, PB250, and PB800 were 10, 12, 19, and 42 nm, respectively, when measurements were performed at 90° (Figure 2D–F and Figure S20A), which is consistent with the *R*_h_ measured at 173°.

**Figure 2:**
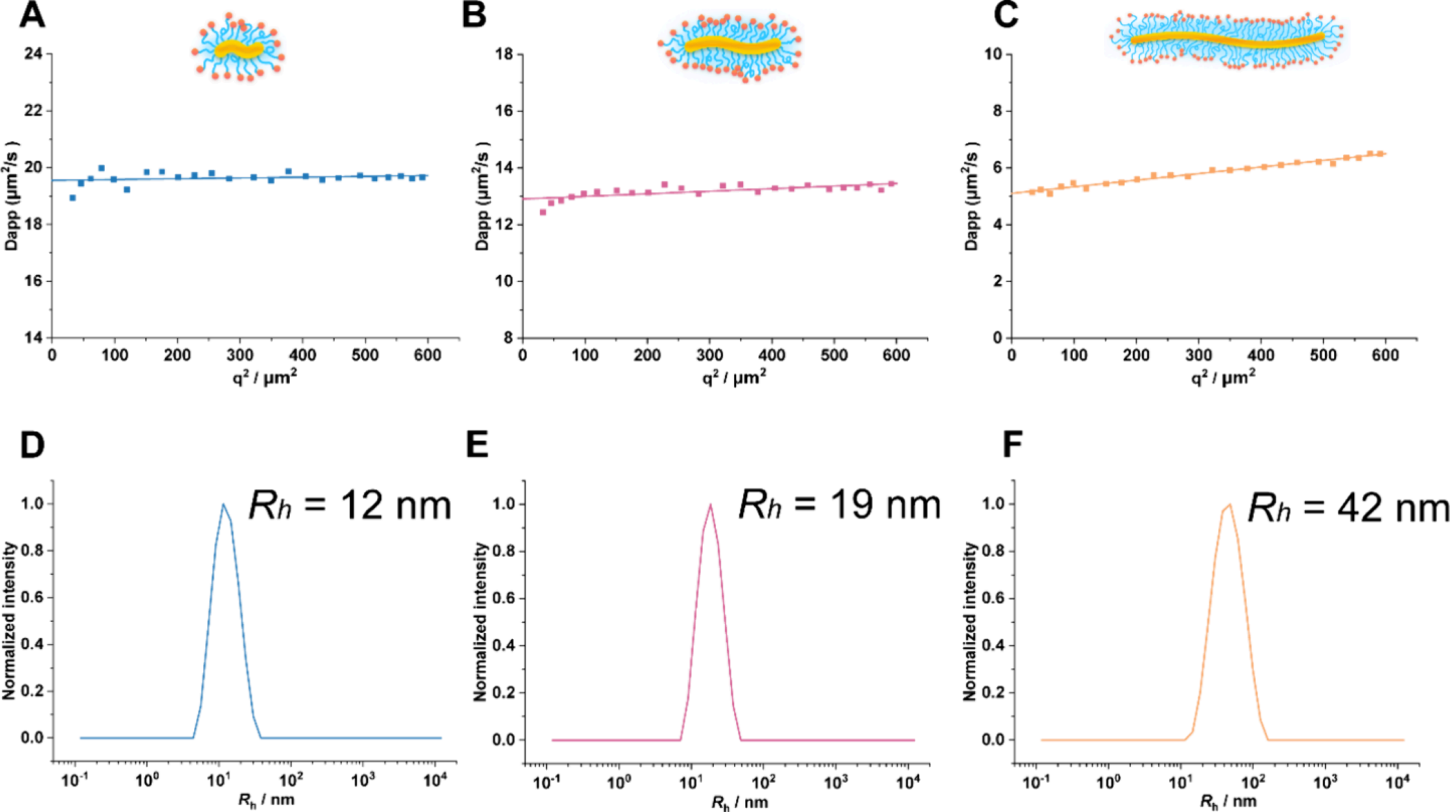
Multiangle DLS analysis of PB100 (A), PB250 (B), and PB800 (C) from 26° to 122° in PBS. Representative size distributions of PB100 (D), PB250 (E), and PB800 at 90° (F).

Additionally, the *R*_g_/*R*_h_ ratios were determined to be 1.0 to 1.4 from **PB50** to **PB800**, indicating the ellipsoidal to worm-like morphology.^60^ The second virial coefficients (*A*_2_) were 1.0 × 10^–6^ mol L/g^2^ for **PB50**, 1.3 × 10^–6^ mol L/g^2^ for **PB100**, 4.2 × 10^–8^ mol L/g^2^ for **PB250**, and 1.5 × 10^–8^ mol L/g^2^ for **PB800** in PBS and reflected good solubility in aqueous solution. Interestingly, we did not observe any differences between commercial and synthesized pLys backbone polypeptides concerning control over pSar side chain length or overall CPB dispersity (Table 1). Furthermore, CD spectroscopy in water showed the transition from a random coil structure of pLys in aqueous solution (minimum at 200 nm, maximum at 220 nm) for all four above-mentioned pLys backbones, confirming the random-coil secondary structure.^61^ Upon synthesis of PBs, the signal for the random coil vanished (Figure S3), which suggests that the random coil pLys backbones extend until the entropic restoring force counterbalances the steric repulsion exerted by the densely packed side chains.^1^ For further analysis and biological evaluation of peptobrushes, we focused on **PB100**, **PB250**, and **PB800**.

The morphology of **PB100**, **PB250**, and **PB800** was visualized using scanning force microscopy (SFM, Figure 3A). Upon analysis of **PB100**, **PB250**, and **PB800** on mica (Figure S22), the average lengths of the PBs were 33, 50, and 125 nm, respectively (Figure 3B). Interestingly, the average widths, ranging from 15 to 19 nm (Figure 3C), and heights from 0.4 to 0.6 nm (Figure S23) of the three PBs were consistent, which is again in line with the comparable pSar side chain lengths of 41–49. These results underscore the high controllability of the synthesis of CPBs based on polypept­(o)­ides. The observed morphologies varied from ellipsoidal to worm-like structures, with average aspect ratios of 1.7, 3.3, and 8.3 (Table 1). These findings were further corroborated by the *R*_g_/*R*_h_ ratio obtained from light scattering measurements, reflecting the diverse structural characteristics of the CPBs.

**Figure 3:**
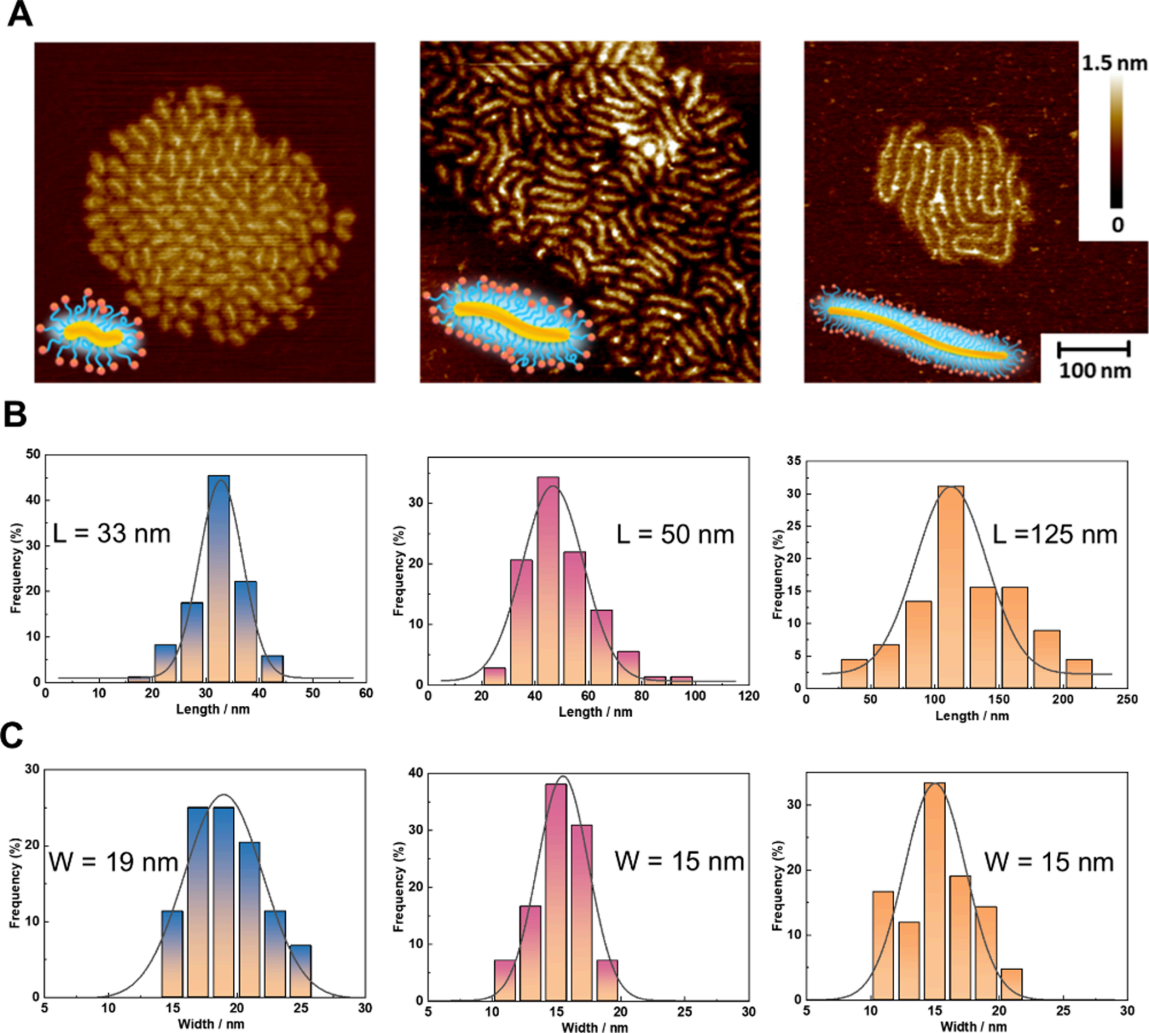
Representative SFM images of **PB100**, **PB250**, and **PB800** (A) and analysis (B and C) on a mica surface. All images have a lateral scale bar of 100 nm and *z*-range of 1.5 nm.

In addition, we investigated the influence of elevated solution temperatures on the size of the PBs. After heating for 24 h at 60 °C, the *R*_h_ of **PB250** was around 19 ± 0.5 nm, without any significant changes (Figure 4A). Following two freezing cycles, the *R*_h_ was around 23 ± 0.7 nm, indicating the thermal stability of PBs in aqueous solution.

**Figure 4:**
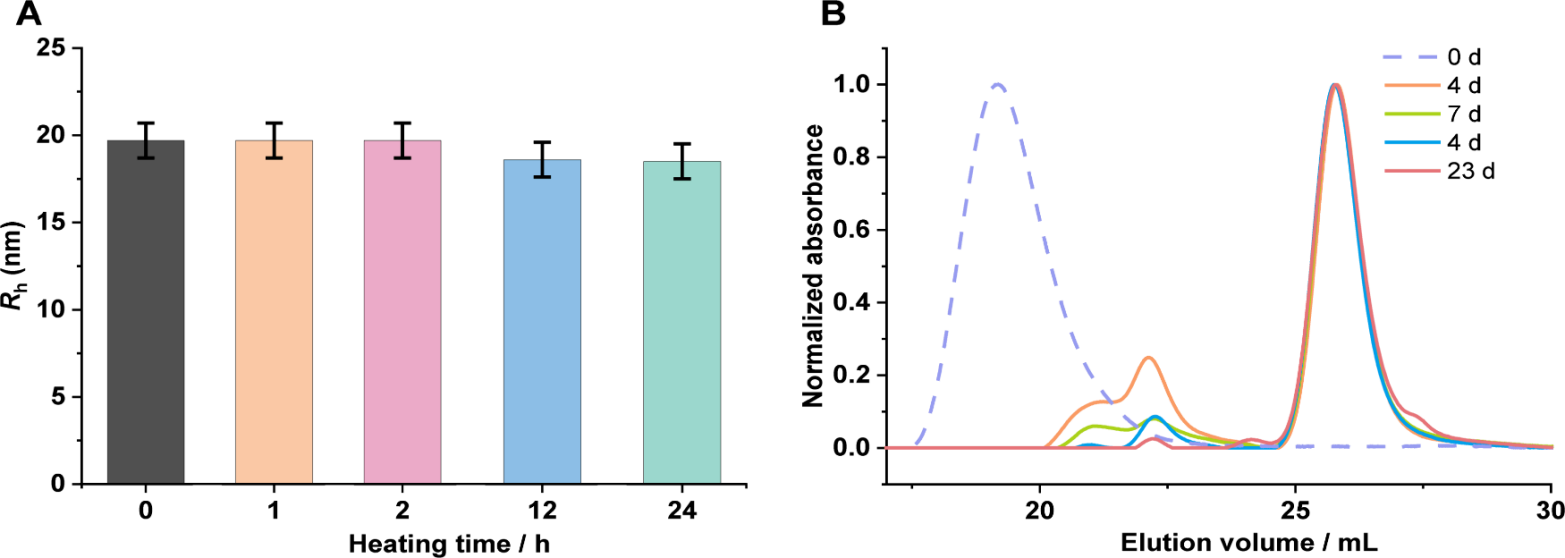
(A) Multiangle DLS measurements for the thermal stability test in terms of average hydrodynamic radius (*R*_h_) of **PB250** in PBS at angles of 26°, 58°, 90°, and 122° (*n* = 5). 1 mg mL^–1^ PB250 in PBS was kept at 60 °C in an oil bath, and its *R*_h_ measurements were recorded at 0, 1, 2, 12, and 24 h. (B) HFIP-SEC monitored the enzymatic degradation of **PB800** with protease coincubation in PBS at 37 °C at different time points. The mass ratio of the enzyme to brush polymer is 3:2.

Since the grafting-from approach makes definitive characterization of side chains usually more complicated than other synthesis strategies, it is not the case for the introduced peptobrushes. Taking advantage of the polypept­(o)­ide nature of the system, enzymatic degradation digesting only the peptide-based backbone while leaving the pSar side chains intact allows for distinct characterization of the latter. pSar-based nanoparticles are stable in blood and display stealth-like nature *in vivo*,^37,39^ as the polypeptide backbone may be accessible for degradation by proteases in *ex vivo* degradation assays. One example is the natural protease B from *Streptomyces griseus*, which is on the one hand rather small (185 amino acids, *R*_h_ ≈ 1.6 nm) and on the other hand known to show broad-spectrum activity in hydrolyzing peptide bonds.^62^ This approach allows confirming the controlled chain growth mechanism known for the Sar-NCA ROP and rendering the NMR and SLS analysis as meaningful. The degradation kinetics of PBs were tracked after incubating with the enzyme in buffer at 37 °C. The degradation product was analyzed by SEC in HFIP. The shift of the initial peak and the appearance of second and third peaks at higher elution volumes indicate the degradation of peptobrushes (Figure 4B and Figure S24). The larger-sized PBs showed faster degradation, and over 95% of **PB800** was degraded within 23 days (Figure 4B). **PB250** exhibited less enzyme resistance compared to **PB100** (Figure S24). As expected, the integrity of pSar was not affected after being exposed to *Streptomyces griseus* proteases. The length of the degraded pSar side chains was determined by HFIP-SEC using polysarcosine standards as reference.^54^

These results highlight a clear structure–function relationship, demonstrating that backbone length and grafting density significantly influence enzymatic accessibility. However, precise independent tuning of grafting density remains challenging due to intrinsic constraints of the grafting-from method. Each lysine residue on the poly-l-lysine backbone inherently acts as an initiation site, and the initiation efficiency is predominantly predetermined during polymerization. Future efforts will thus focus on developing alternative synthetic strategies or protective-group chemistries to enable precise control over the grafting density, independent of backbone length. Such advancements will facilitate systematic studies investigating how individual structural parameters (e.g., aspect ratio, grafting density, and chain flexibility) influence enzymatic degradation kinetics at equivalent molar concentrations. Extending these degradation studies to more physiologically relevant *in vivo* models (e.g., rodents) will be essential for translating these findings into practical biomedical applications.

Furthermore, as shown in Table 1, the repeating units of pSar sides are calculated to 44, 54, and 35 with dispersity values between 1.03 and 1.04, which underlines the controlled chain growth from the pLys backbone. More importantly, this data, in comparison to the DP values by ^1^H NMR, allowed us to calculate the grafting density to be 93% (**PB100**), 89% (**PB250**), and 85% (**PB800**). Notably, even for the larger-sized brush **PB800** (*M*_w_ ≈ 3,000,000 g mol^–1^), almost each lysine side chain has initiated a pSar side chain, and the ROP proceeds under highly controlled conditions.

The chemical versatility of an azide group present at each pSar side chain allows for fluorescent labeling, thus enabling us to track their behaviors under biological conditions. **PB100**, **PB250**, and **PB800** labeled with Alexa Fluor 647 were examined using FCS both in PBS and 90% (vol%) human serum (Table 1 and Figure S2). The aforementioned peptobrushes exhibited altogether a slight difference in hydrodynamic radius (around 1 nm) at 1 h exposure in human serum (Figure S1B) compared to the analogues in PBS (Figure S1A), which is in the error range of the measurement itself and therefore insignificant.^63^ This data indicate the physiological stability and protein absorption resistance of all three PBs, with respect to the size and the absence of aggregation. Additionally, extended incubation studies up to 24 h again showed the long-term colloidal stability of these peptobrushes in human serum (Figure 5).

**Figure 5:**
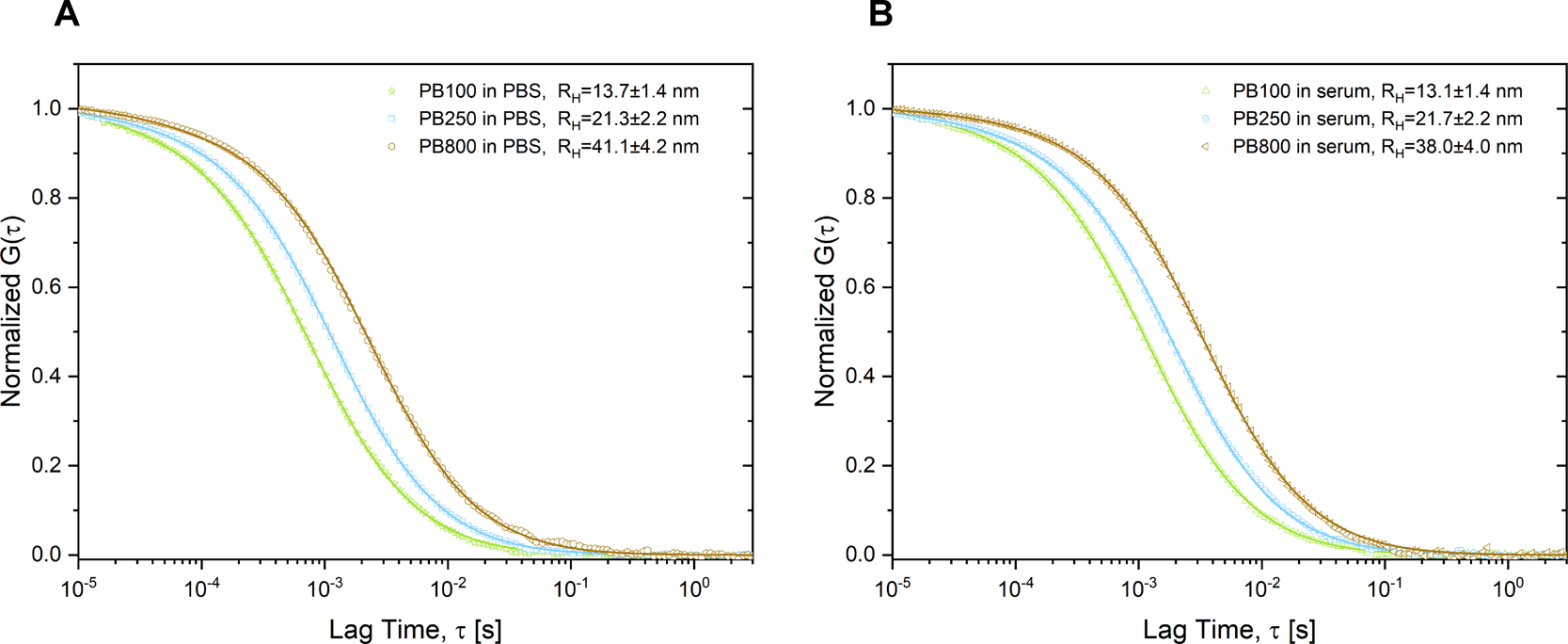
Autocorrelation analysis of Alexa Fluor 647 labeled **PB100**, **PB250**, and **PB800** after 24 h of incubation in PBS (A) and human serum (B) by FCS.

To further confirm the biocompatibility of polypept­(o)­ide-based brush polymers, we evaluated the cytotoxicity of the three synthesized brush polymers (**PB100**, **PB250**, and **PB800**) using HeLa cells, Raw264.7 cells, and Jurkat cells at final concentrations ranging from 0.5 to 10 μg mL^–1^. As the MTT assays in Figure S26 show, cell viability remained over 80% after 24 h exposure for both HeLa cells and Jurkat cells, regardless of the applied brush polymer dose. While around 70% of living Raw264.7 cells were detected across all brushes, **PB800** demonstrated a slight increase in cell viability (%) compared to **PB100** and **PB250** at the same dose. Overall, all these brushes showed low cytotoxicity and good biocompatibility.

Considering the stealth-like nature of pSar and the steric repulsion in CPBs, we studied the interaction of such peptobrushes with different cell lines. After 4 h exposure, there was no detectable cellular association for all peptobrushes in HeLa (<10%, Figure 6A2, B2), Raw264.7 cells (<5%, Figure 6A3, B3), and Jurkat cells (<10%, Figure 6A4, B4). Further, PBs were incubated in heat-inactivated FBS for 1 h before application to cells for 4 h. While serum pre-exposure induced significant changes in cellular internalization of nanoparticles (e.g., mRNA lipid nanoparticles),^64^ no statistical alternations were detected among all PBs, regardless of the cell lines (Figure 6).

**Figure 6:**
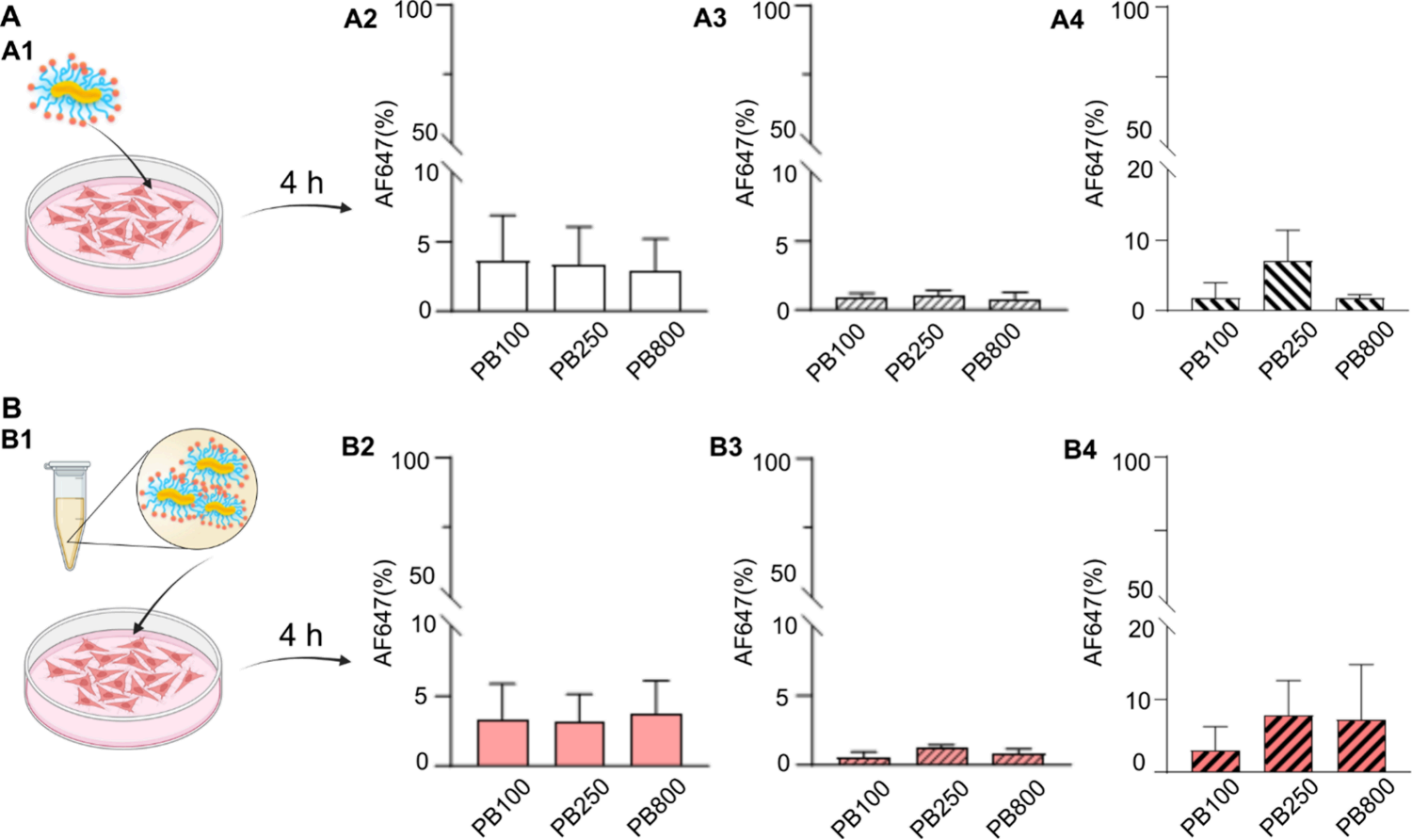
Cellular association (%) of peptobrushes **PB100**, **PB250**, and **PB800** in HeLa (A2, B2), Raw264.7 (A3, B3), and Jurkat cells (A4, B4). Peptobrushes in PBS were directly applied onto cells (A1) or preincubated with FBS (B1) for 1 h at 37 °C before being applied onto cells. After 4 h exposure, cells were harvested and quantified by flow cytometry. All experiments were performed in triplicate (*n* = 9). Statistical significance was evaluated using two-way ANOVA with multiple comparison correction. A *p*-value of <0.05 was considered significant.

Furthermore, we evaluated the performance of these peptobrushes in a more complex and dynamic condition following intravenous injection into zebrafish embryos. As Figure 7 shows, there was no significant difference in the circulation time in zebrafish embryos within 72 hpi (hours postinjection) among all the peptobrushes studied in this research. Within the first 4 h postinjection (hpi), around 25% of peptobrushes were cleared from the circulation, while no dramatic decrease in the relative mean fluorescence intensity was detected within the following 3 days. By 72 hpi, around 50% of peptobrushes remained in the bloodstream of zebrafish embryos. Taken together, the circulation in zebrafish embryos is independent of size, shape, and morphology in the context of PBs investigated in this study. This result can be attributed to the developmental stage of the zebrafish liver, which begins to develop at around 48 hpf and matures by approximately 72 hpf, coinciding with the time frame of our observations. Previous studies have shown that nanoparticles smaller than 10 nm are rapidly cleared by the kidney, while those larger than 50 nm are quickly removed by Kupffer cells.^65^ However, since the zebrafish liver was only maturing during our experimental period, our study did not prominently observe size-dependent differences in circulation time.

**Figure 7:**
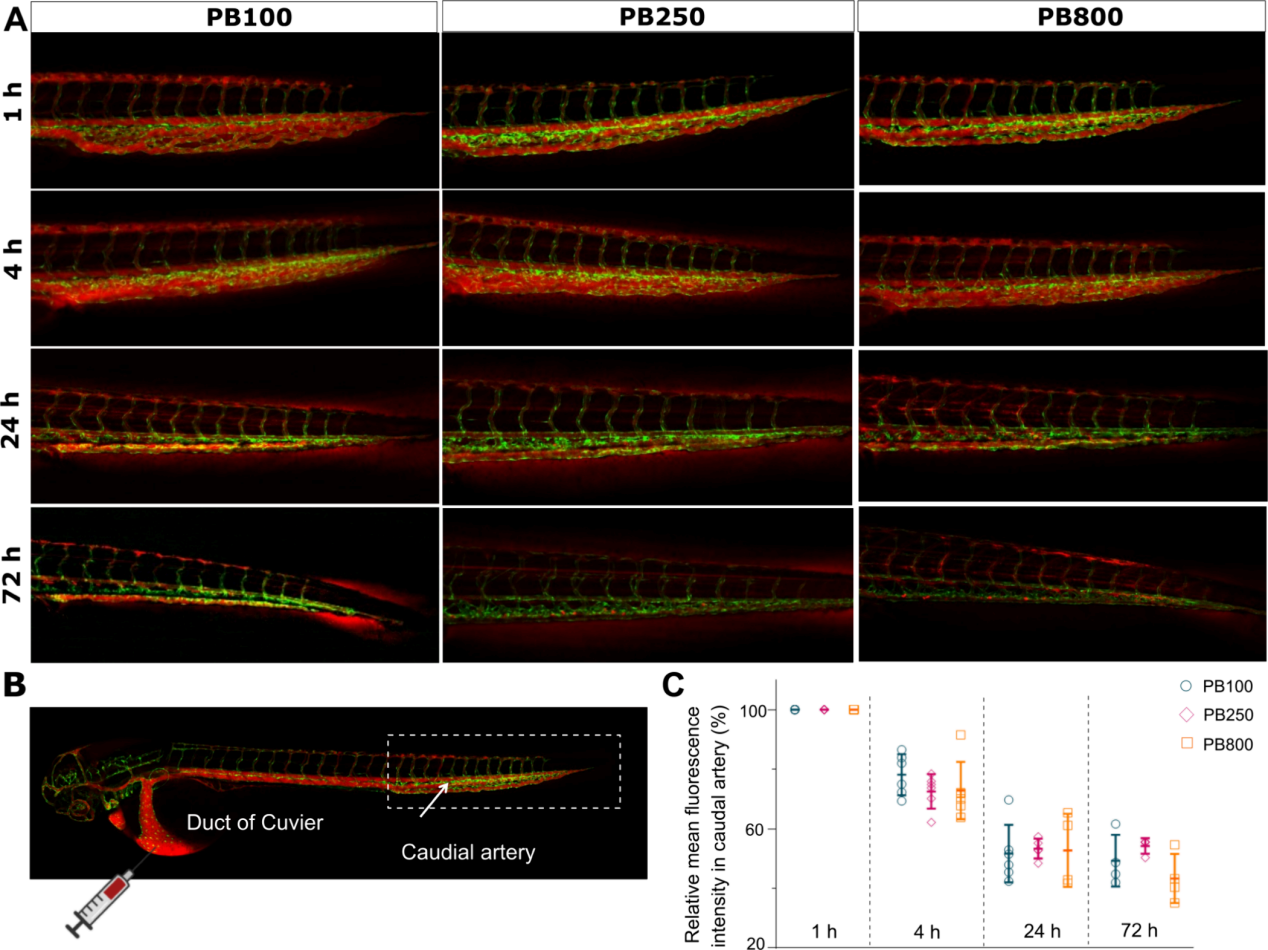
(A) Representative images of zebrafish embryos at 1, 4, 24, and 72 hpi. (B) Two days post fertilization zebrafish embryos were injected with 1 nL per fish into the Duct of Cuvier (at a concentration of 2 mg mL^–1^ for **PB100**, 5 mg mL^–1^ for **PB250**, and 8 mg mL^–1^ for **PB800**). The caudal artery of the embryos was used for quantification of circulation. (C) Relative mean fluorescence intensity in the caudal artery was measured, with 1 h imaging for calculation and analysis. Significance between the means of three groups was tested using one-way ANOVA with the software GraphPad Prism 8. A *p*-value of <0.05 was considered significant.

## Conclusions

Controlled synthesis and detailed characterization of polypept­(o)­ide-based CPBs were achieved in this study, addressing the central challenge of creating biocompatible and strongly biodegradable systems. By introducing polypept­(o)­ide-based CPBs, which combine the biodegradability of pLys with the advantageous properties of pSar, we provide a viable alternative to traditional nondegradable CPBs with hydrocarbon backbones. The developed CPBs remain stable in various biological solutions across different conditions but degrade in the presence of protease. Moreover, these peptobrushes display high protein resistance and long circulation times in zebrafish embryos (up to 3 days), independent of the size, shape, and morphology. Consequently, the CPBs described here, based on endogenous amino acids, not only enable the production of polymeric nanoparticles with precise control over size, morphology, and the possibility for surface modification but also provide a promising foundation for multiple biomedical applications. By utilizing the well-defined polysarcosine side chains with stealth-like properties, we established a highly demanded alternative to currently used strategies to overcome disadvantages associated with polymeric systems in biomedicine.

## Supplementary Material



## Notes

Representative peptobrush syntheses conditions; FCS analysis; SEC analysis; CD spectroscopy analysis; FT-IR spectroscopy analysis; ^1^H NMR-data; DOSY-NMR; DLS analysis; SFM analysis; peptobrush degradation assay analysis; MTT assay analysis and additional information (PDF)
