# Supplementary material for: Polypept(o)ide Based Biodegradable Cylindrical Polymer Brushes: Controlling Size, Shape, Surface Functionality, and Stability
Source: ACS Appl Mater Interfaces. 2025 Oct 2;17(46):62969–82. doi: 10.1021/acsami.5c15018 (PMC12635966; doi:10.1021/acsami.5c15018)
Supplement: Supplementary file 1 [file am5c15018_si_001.pdf]

Supporting Information

Polypept(o)ide based Biodegradable Cylindrical Polymer Brushes: Controlling Size, Shape, Surface Functionality and Stability

Christine Ilona Seidl‡[1,2], Bonan Zhao‡[1], Xinye Gao‡[1], Rüdiger Berger[3], Lin Jian[3], Kaloian Koynov[3], Meike Gangluff[4,5], Rivka Fontijn[1], Lu Su[1], Jeroen Bussmann[1], Heyang Zhang[1], Matthias Barz[1,2]*

[1]Leiden Academic Center for Drug Research (LACDR), Leiden University, Einsteinweg 55, 2333CC Leiden, The Netherlands.

[2]Department of Dermatology, University Medical Center of the Johannes Gutenberg University Mainz, Langenbeckstraße 1, 55131 Mainz, Germany

[3]Physics at Interfaces, Max Planck Institute for Polymer Research, Ackermannweg 10, 55128 Mainz, Germany

[4]Department Chemie, Johannes Gutenberg University Mainz, Duesbergweg 10-14, 55131 Mainz, Germany

[5] BioNTech SE, An der Goldgrube 12, 55131 Mainz, Germany

‡ The authors contribute to the work equally.

Corresponding Author: Matthias Barz (m.barz@lacdr.leidenuniv.nl)

Tables of Contents

Page

Representative Peptobrush syntheses conditions 3

Representative FCS analysis 4

Representative SEC analysis 6

Representative CD spectroscopy analysis 7

Representative FT-IR spectroscopy analysis 8

Representative ^{1}H NMR-data 10

Representative DOSY-NMR 14

Representative DLS analysis 16

Representative SFM analysis 20

Representative Peptobrush degradation assay analysis 22

Representative MTT assay analysis 23

Additional Information 24

Supplementary Tables and Figures:

Representative Peptobrush syntheses conditions:

Table S1: Peptobrush syntheses conditions.

**Polymer Brush**	**Polyinitiator**	**Sar-NCA feeding ratio (*eq.*)**	**DIPEA feeding ratio (*eq.*)**	**Solvents**	**Reaction temperatures (°C)**	**Reaction times (d)**
PB50, p(L)Lys_{50}-*g*-$pSar_{40}(N_3)$	$pLys_{50}$·TFA	40	1.2	Dry DMF	10	4
$PB100_{Short}$, p(L)Lys_{100}-*g*-$pSar_{25}(N_3)$	$pLys_{100}$ ·TFA (synthesized)	25	1.2	Dry DMF	10	3
PB100, p(L)Lys_{100}-*g*-$pSar_{40}(N_3)$	$pLys_{100}$·TFA	40	1.2	Dry DMF	10	5
PB250, p(L)Lys_{250}-*g*-$pSar_{40}(N_3)$	$pLys_{250}$·TFA	40	1.2	Dry DMF	10	4
PB300, p(L)Lys_{300}-*g*-$pSar_{45}(N_3)$	$pLys_{300}$ ·TFA (synthesized)	40	1.2	Dry DMF	10	5
PB430, p(L)Lys_{430}-*g*-$pSar_{76}(N_3)$	$pLys_{430}$ ·TFA (synthesized)	80	1.2	Dry DMF	10	8
PB800, p(L)Lys_{800}-*g*-$pSar_{40}(N_3)$	$pLys_{800}$·TFA	40	1.2	Dry DMF	10	5
PB900, p(L)Lys_{896}-*g*-$pSar_{79}(N_3)$	$pLys_{896}$ ·TFA (synthesized)	80	1.2	Dry DMF	10	8

Representative FCS analysis:

Table S2. Analysis of Alexa Fluor647 labeled PB100, PB250 and PB800 in PBS and human serum (vol%, 90%) by FCS within 24 h.

Polymer	t (h)	In PBS			In serum	Aggregates	
		R_h (nm)	Dye per brush	Free dye (%)	R_h (nm)	PBS	serum
PB100	0	12.9	5.0	5.6	12.7	No	No
	1	12.9	/	/	13.3		
	4	12.9	/	/	12.8		
	24	13.7	/	/	13.1		
PB250	0	19.1	10.3	3.0	20.3	No	No
	1	19.1	/	/	21.1		
	4	19.1	/	/	20.1		
	24	21.3	/	/	21.7		
PB800	0	39.6	20.4	3.5	36.6	No	No
	1	39.6	/		38.8		
	4	39.6	/		37.8		
	24	41.1	/	/	38.0		

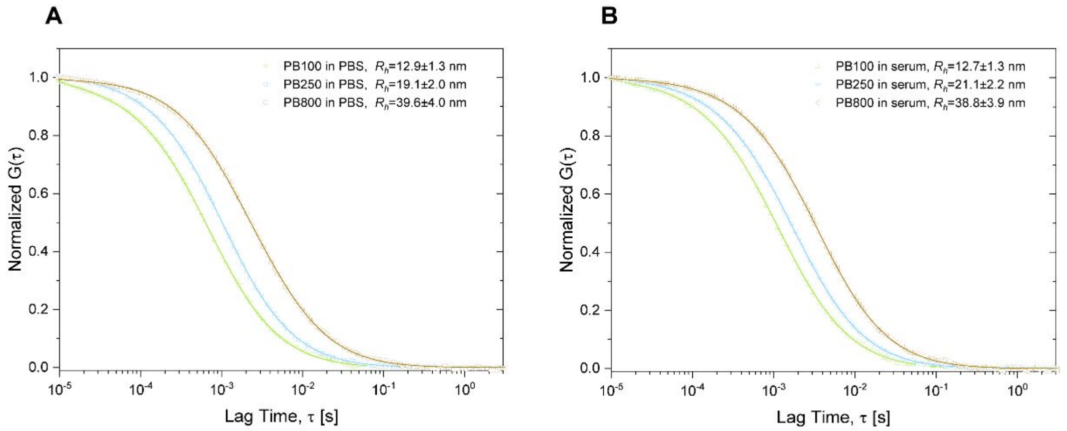

Figure S1. Autocorrelation analysis of Alexa Fluor647 labeled PB100, PB250 and PB800 after 1 hour of incubation in PBS (A) and human serum (B) by FCS.

Representative SEC analysis:

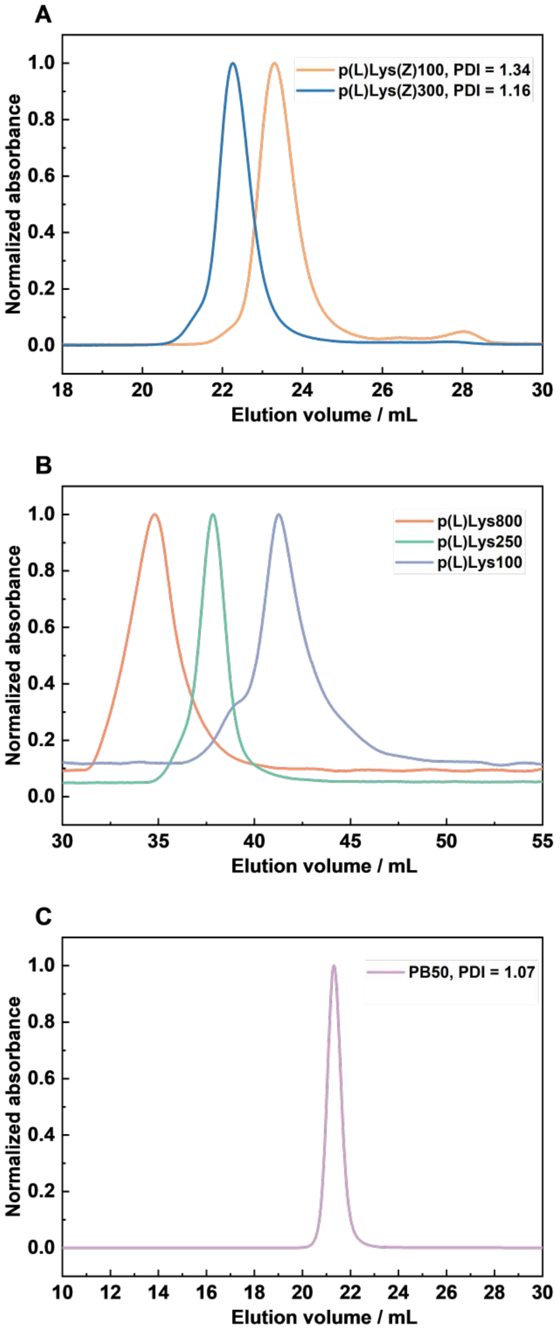

Figure S2. Representative size-exclusion chromatography (SEC) curves of house-synthesized $pLys(Z)_{100}$ and $pLys(Z)_{300}$ backbones in HFIP (A). Notably, no bimodal distributions indicative of secondary structures were observed with increasing chain length, consistent with findings reported by Huesmann et al. (Macromolecules, 2014); Commercial $pLys_{100}$, $pLys_{250}$ and

$pLys_{800}$ backbones in PBS (B) (Provided by Alamanda Polymers, Inc.); PB50 polymer brush in HFIP (C).

Representative CD spectroscopy analysis:

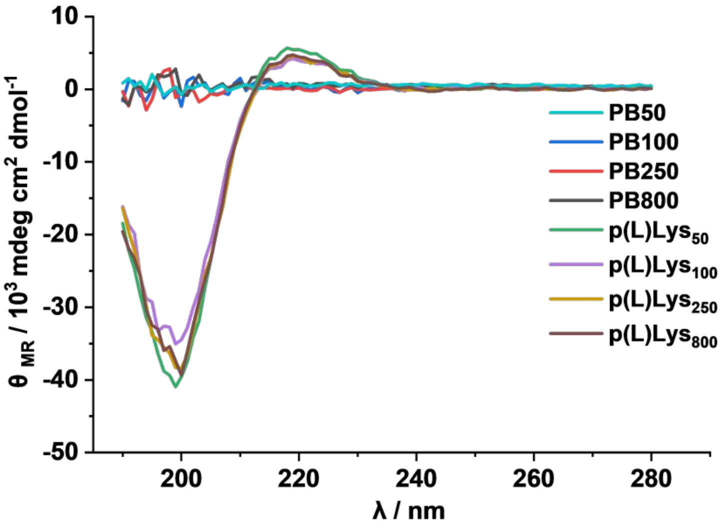

Figure S3. CD spectroscopy of pLys backbone and peptobrush polymers in MQ water.

Representative FT-IR spectroscopy analysis:

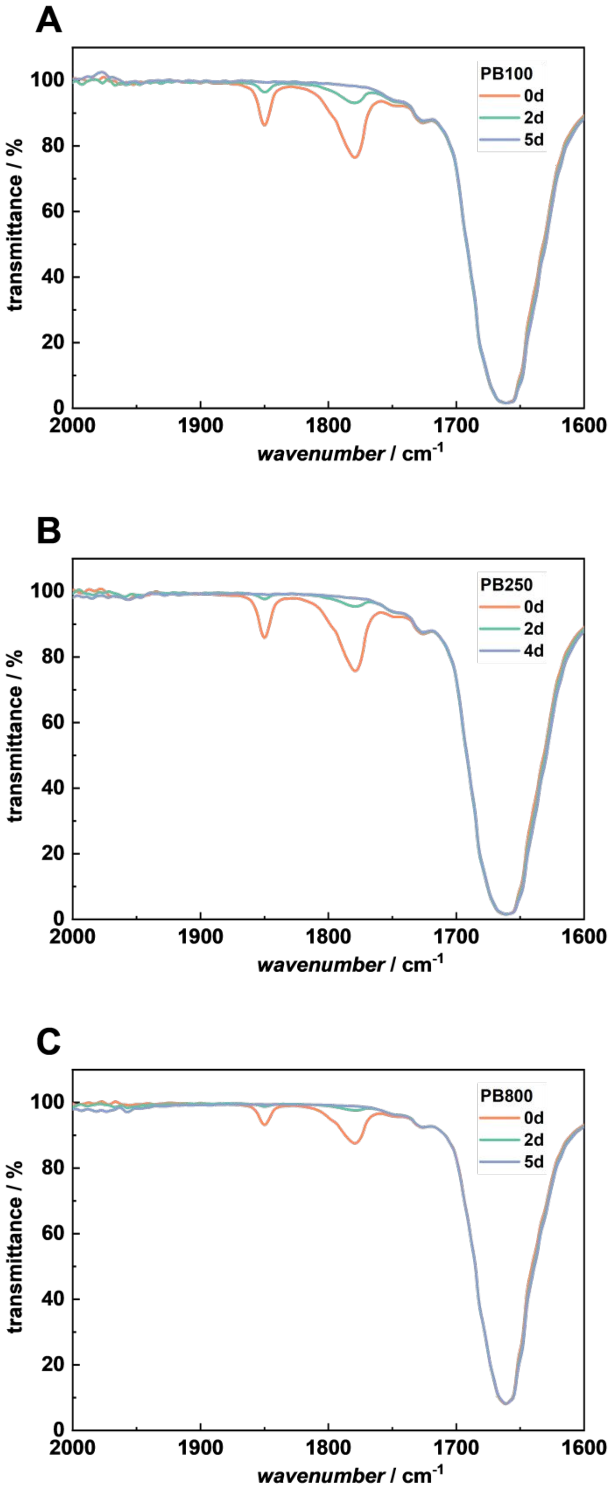

Figure S4. FT-IR spectroscopy monitored the kinetic and confirmed complete polymerization of Sar-NCA for PB100 (A), PB250 (B) and PB800 (C) by the disappearance of carbonyl peaks at 1858 and 1788 cm^{-1}.

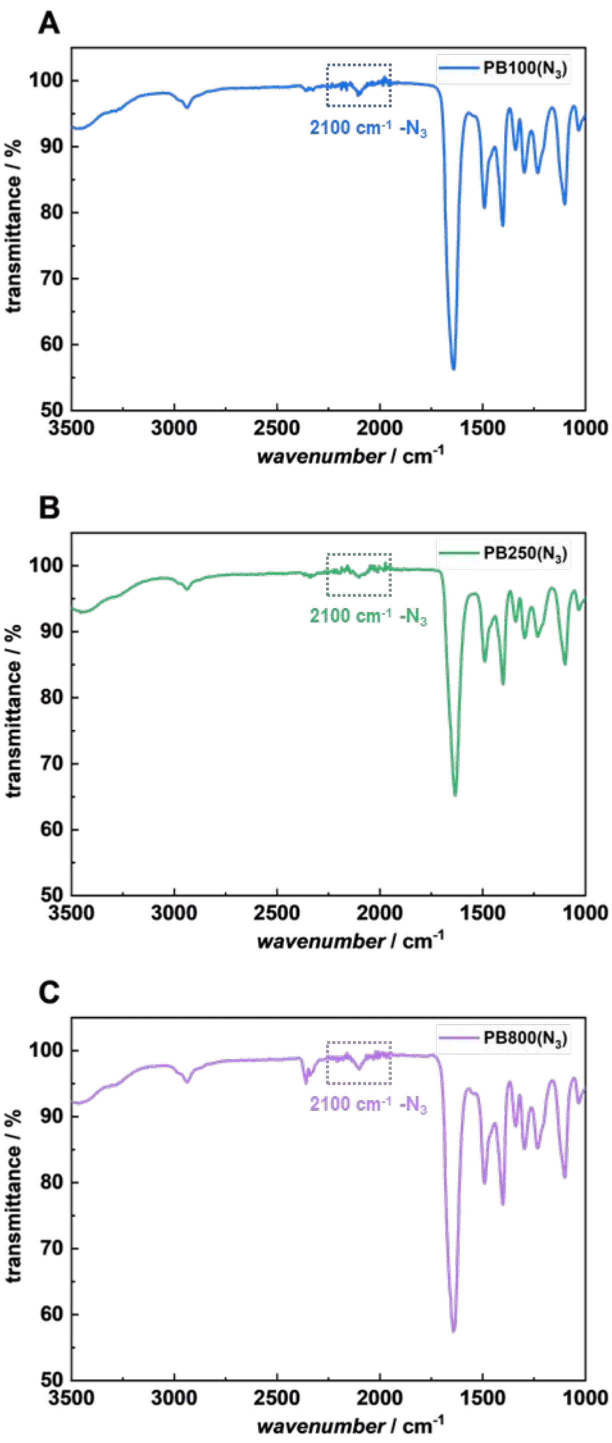

Figure S5. FT-IR spectrum of PB100(N_3) (A), PB250(N_3) (B) and PB800(N_3) (C) confirming the integrity of the azide moiety in the terminal brush architecture at 2100 cm^{-1}.

Representative ^{1}H NMR-data:

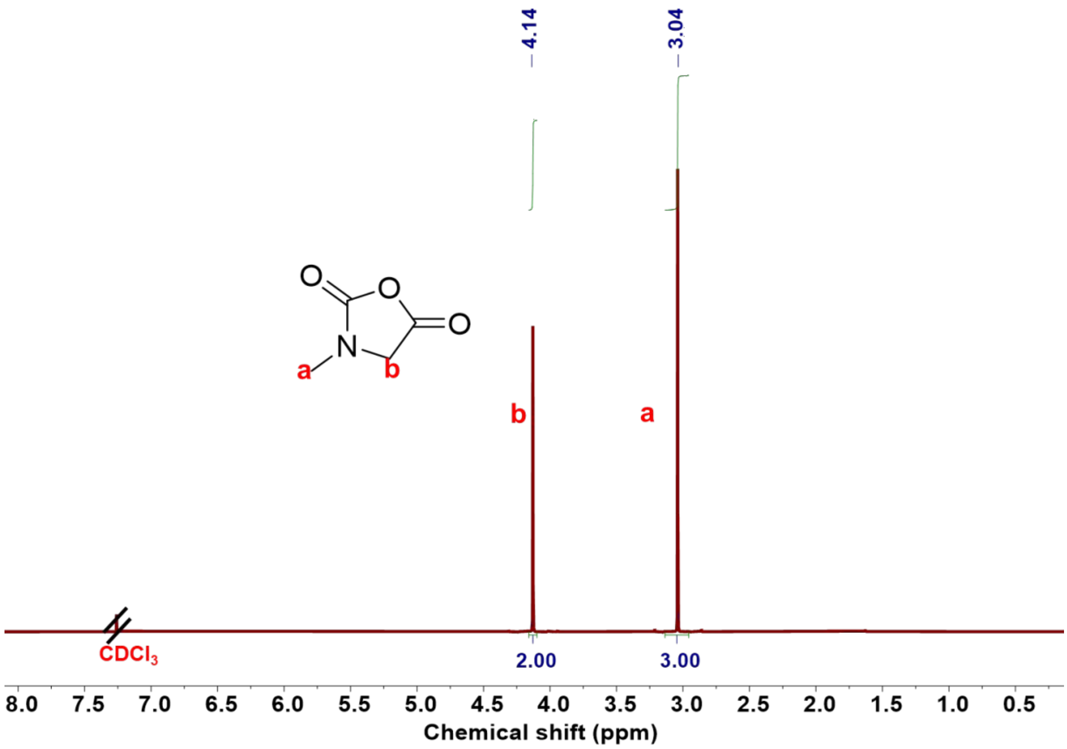

Figure S6. ^{1}H NMR analysis of Sar-NCA in $CDCl_3$.

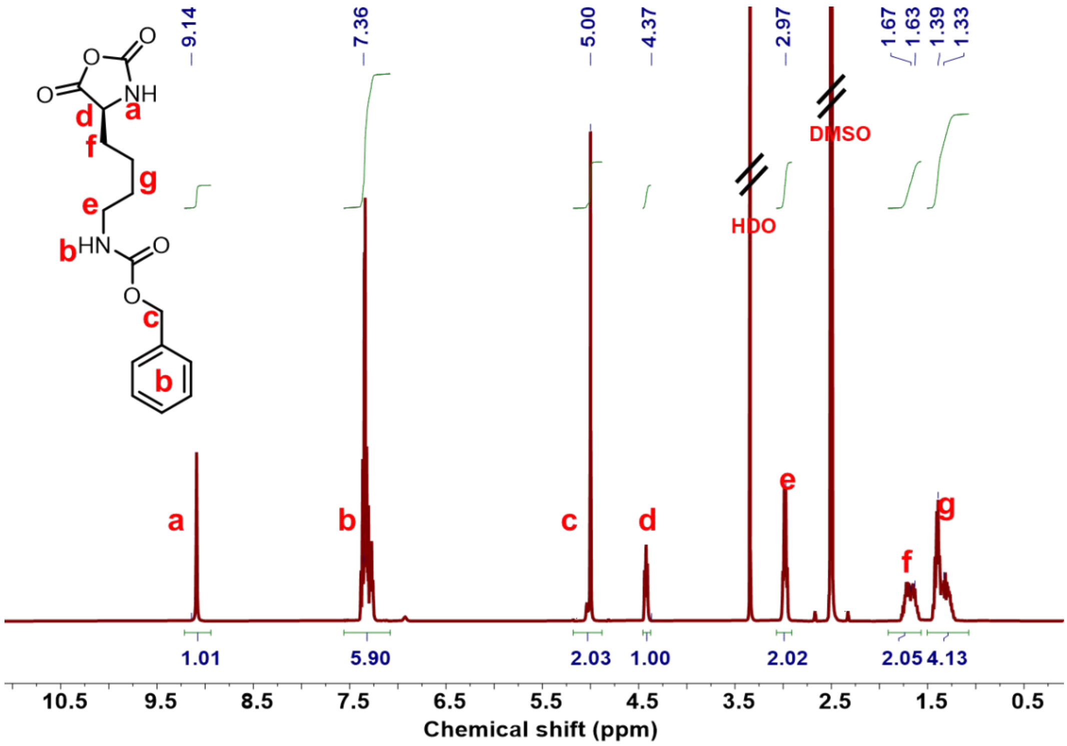

Figure S7. ^{1}H NMR analysis of Lys(Z)NCA in DMSO-d_6.

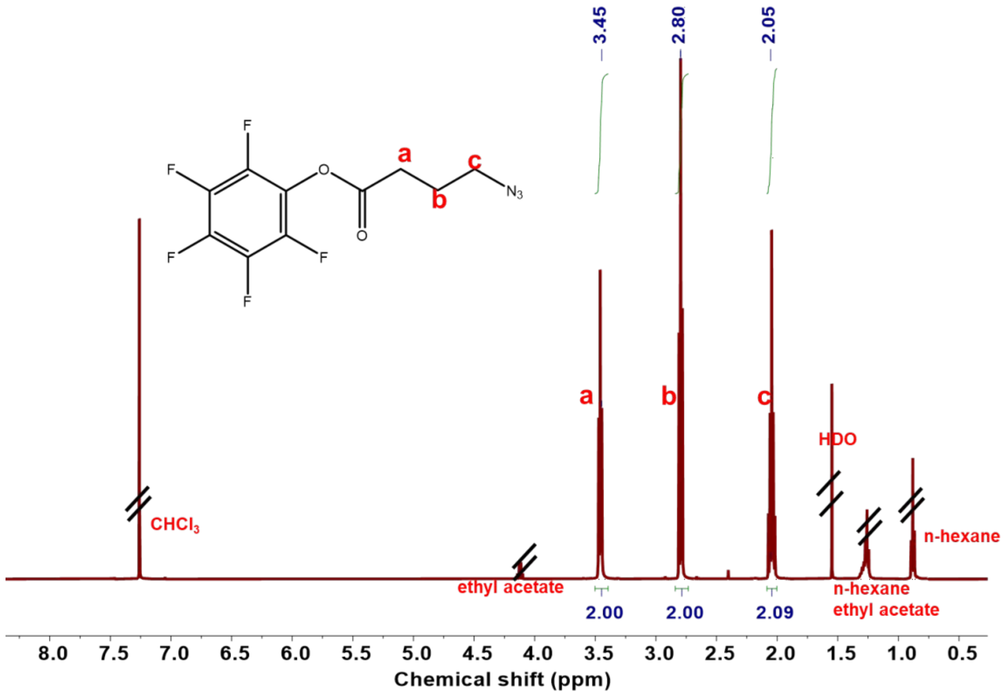

Figure S8. ^{1}H NMR analysis of Azido-Butyric Acid Pentafluorophenyl Ester $CDCl_3$.

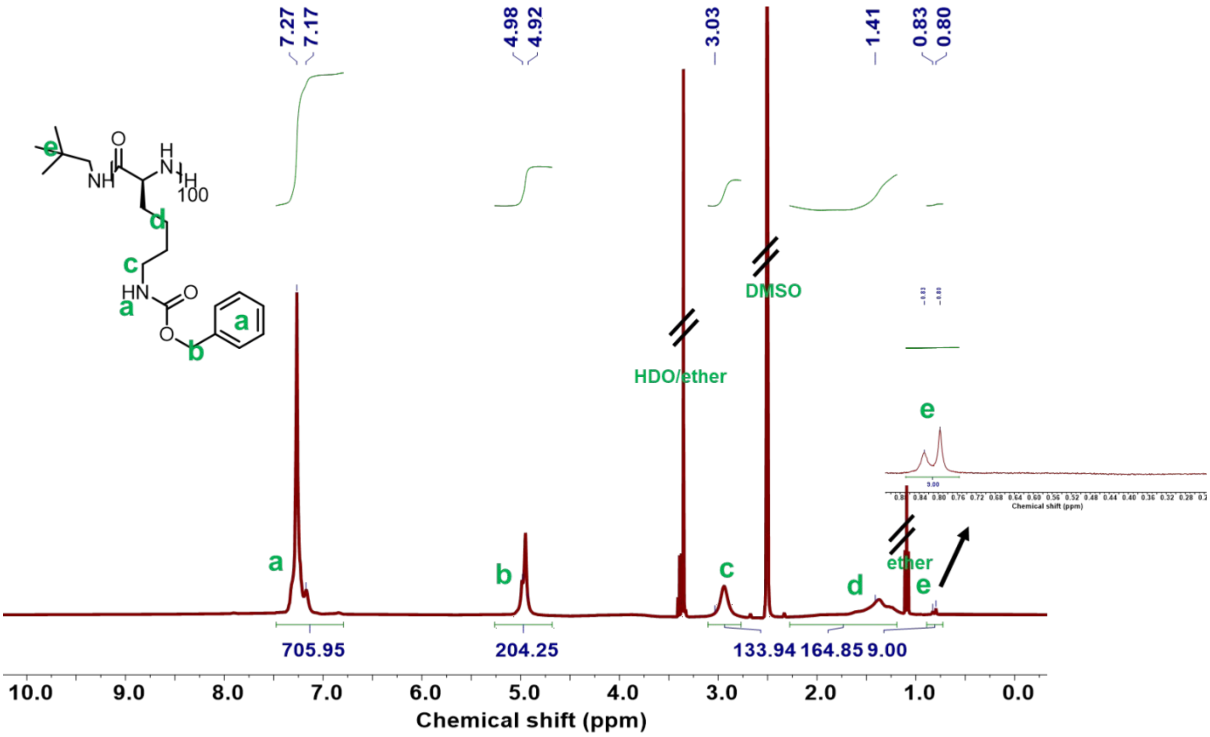

Figure S9. ^{1}H NMR analysis of pLys(Z)$_{100}$ in DMSO-d_6.

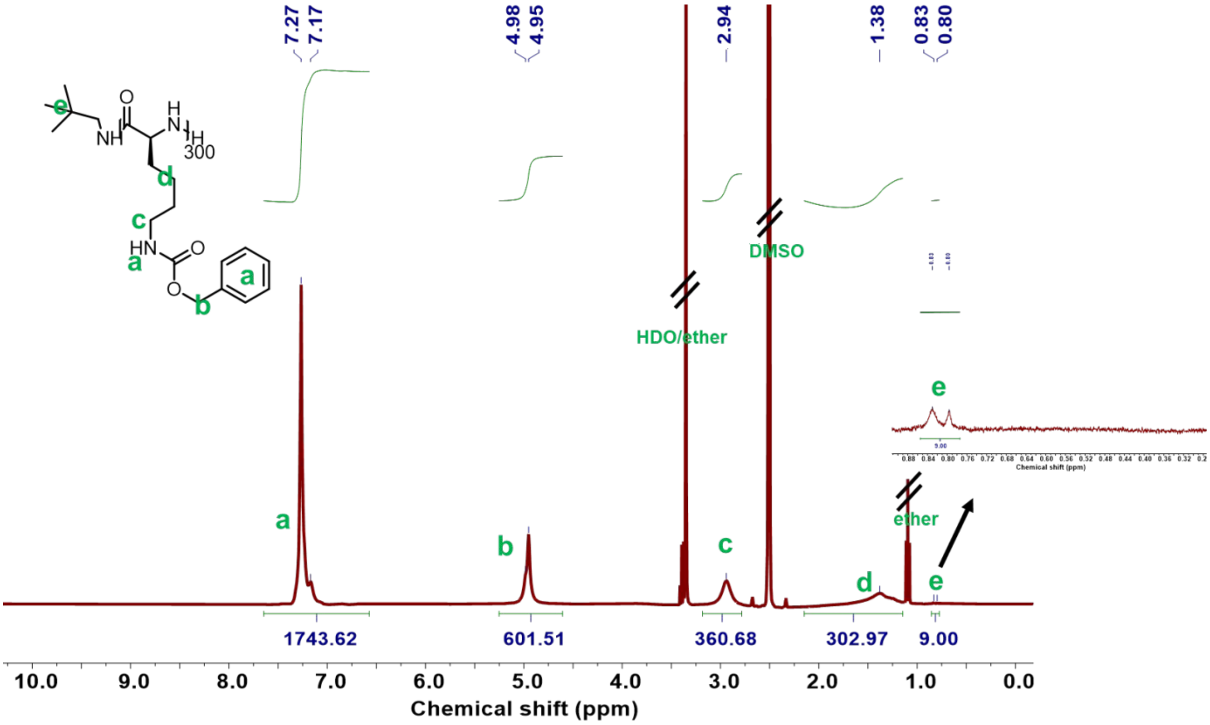

Figure S10. ^{1}H NMR analysis of pLys(Z)$_{300}$ in DMSO-d_6.

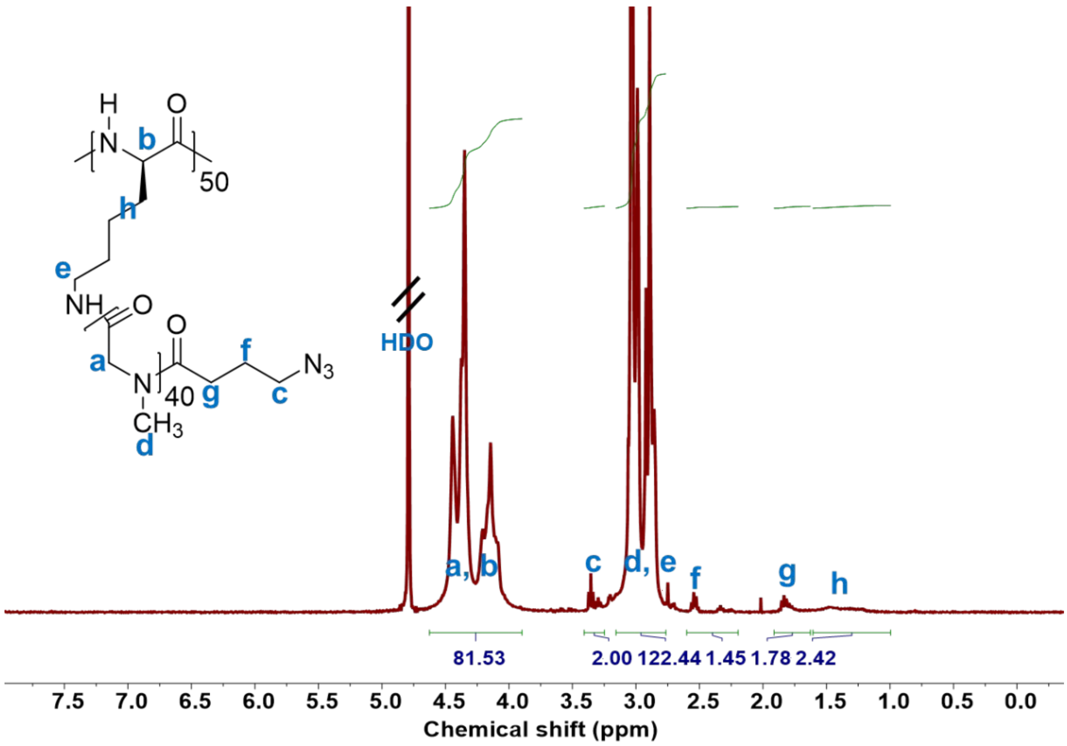

Figure S11. ^1H NMR analysis of PB50 in D_2O.

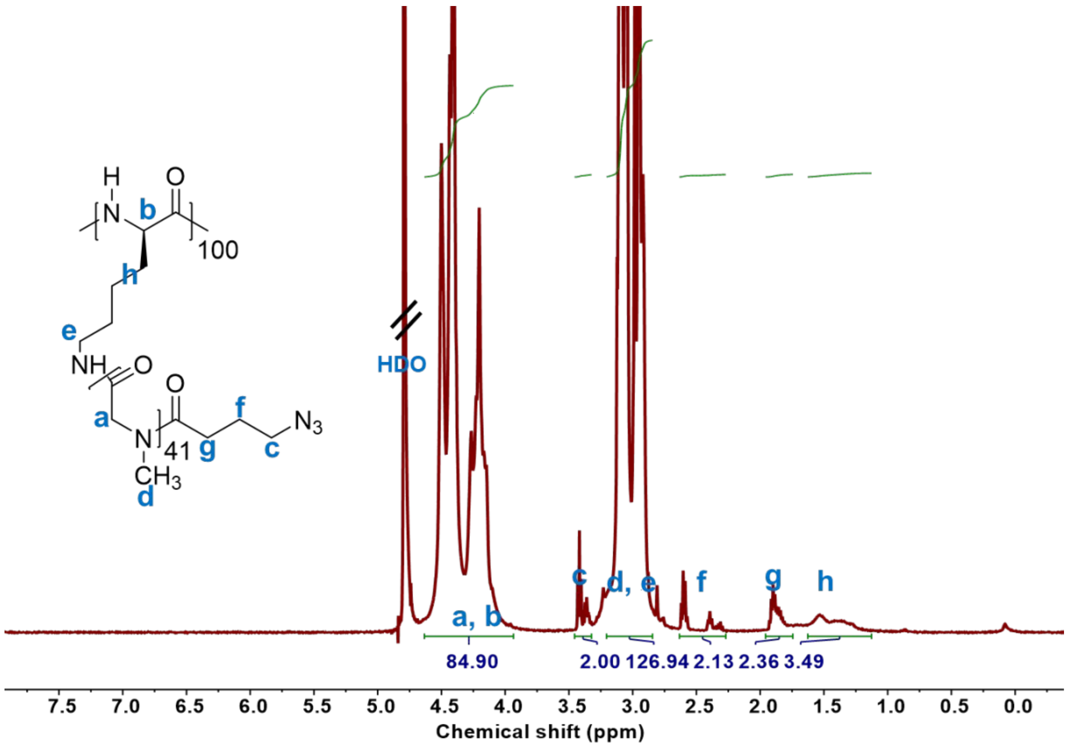

Figure S12. ^1H NMR analysis of PB100 in D_2O.

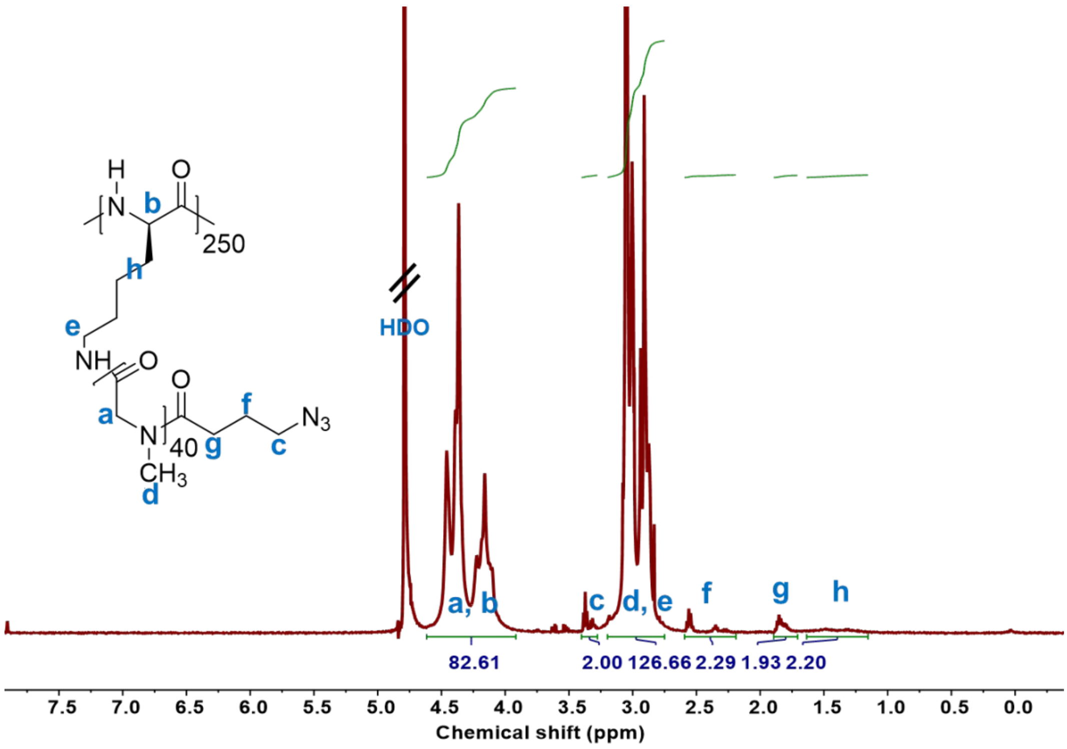

Figure S13. ^{1}H NMR analysis of PB250 in D_2O.

Representative DOSY-NMR:

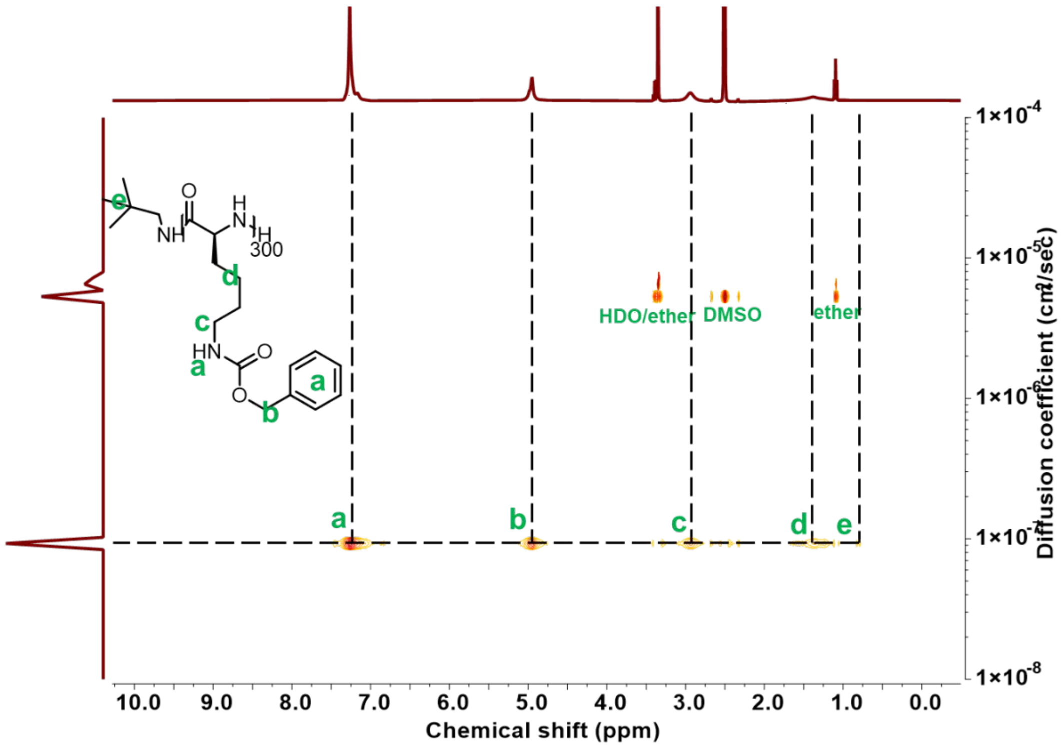

Figure S14. DOSY-NMR analysis of pLys(Z)$_{300}$ in DMSO-d_6.

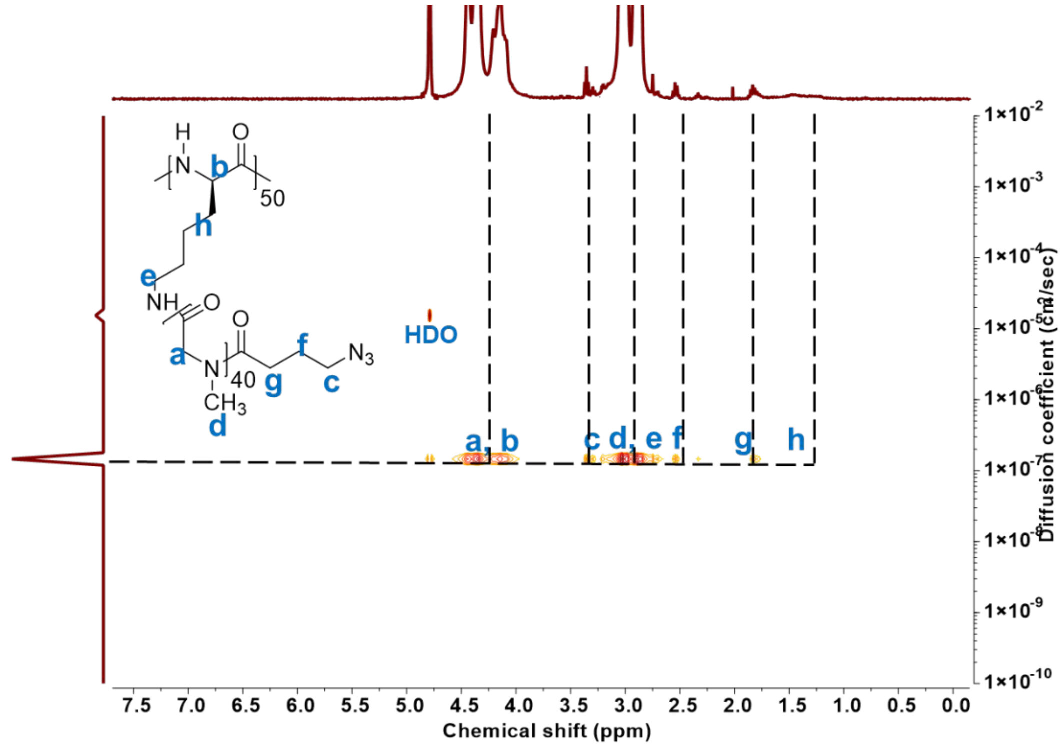

Figure S15. DOSY-NMR analysis of PB50 in D_2O.

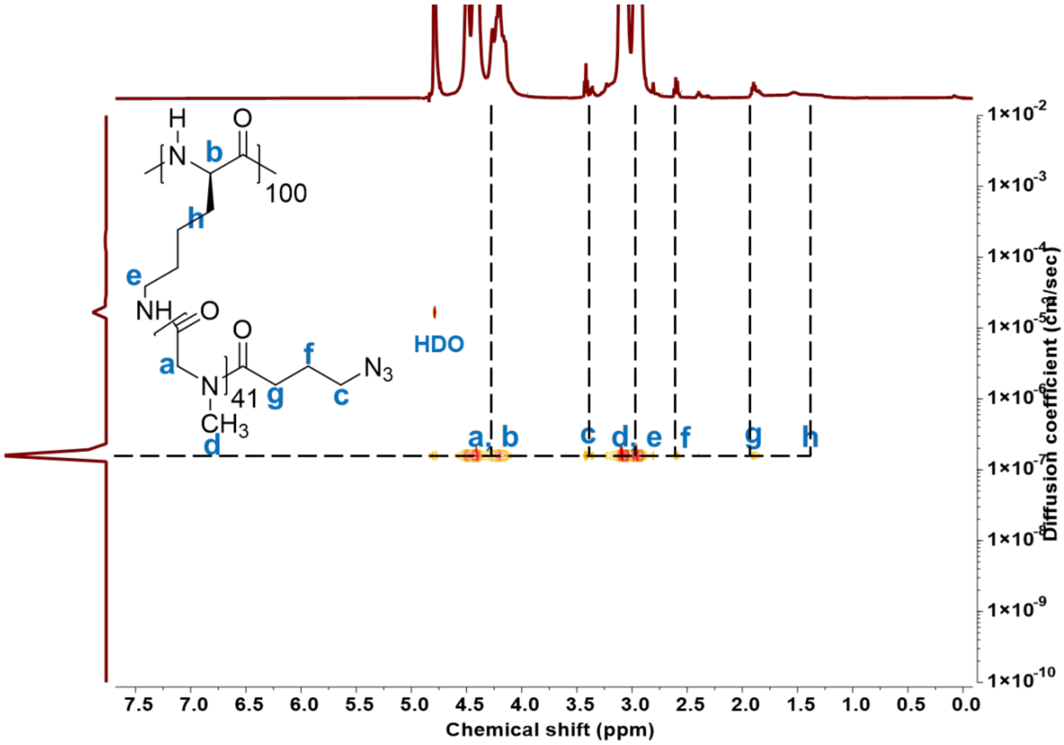

Figure S16. DOSY-NMR analysis of PB100 in D_2O.

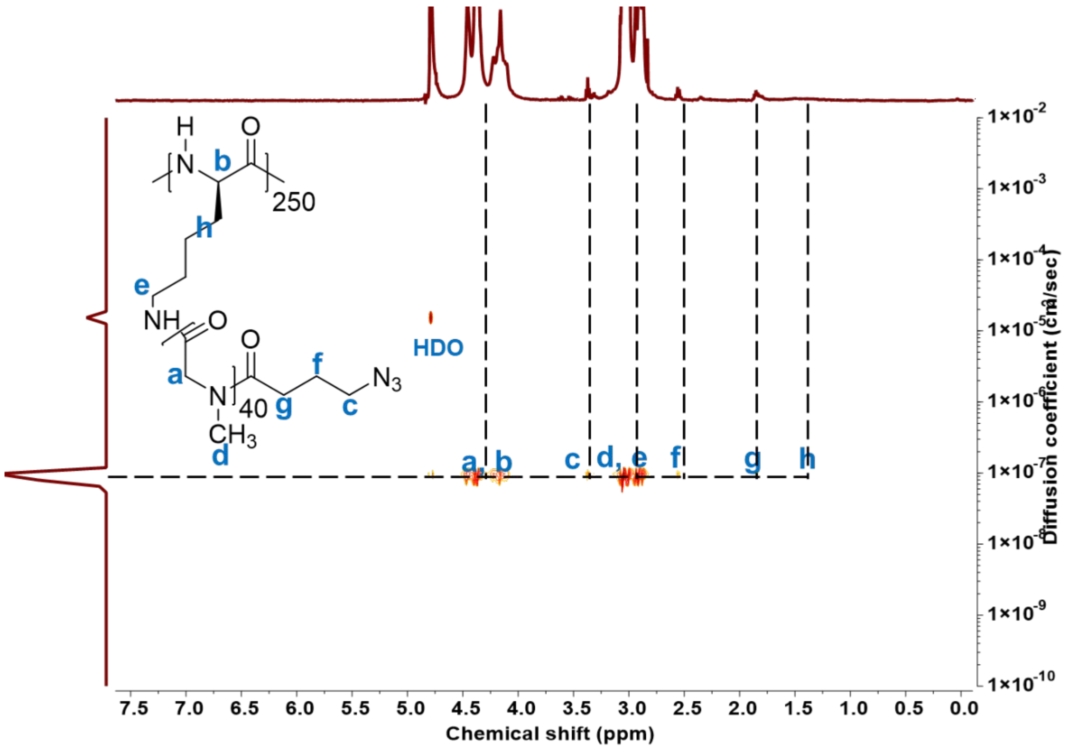

Figure S17. DOSY-NMR analysis of PB250 in D_2O.

Representative DLS analysis:

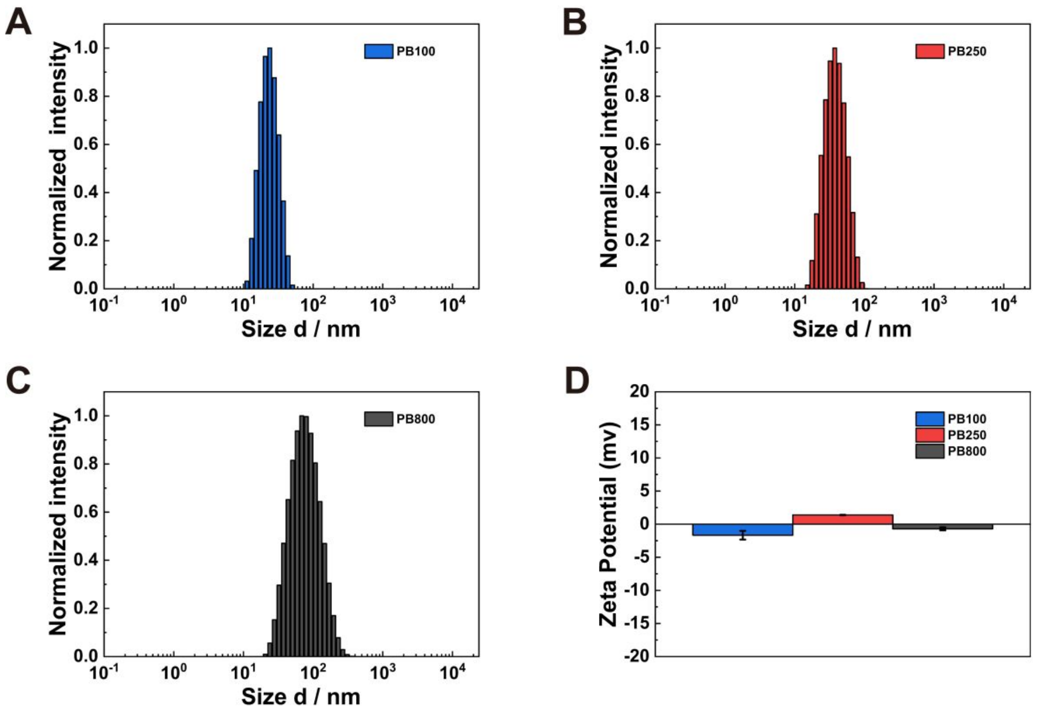

Figure S18. Size distribution of PB100 (A), PB250 (B) and PB800 (C) measured at 173° and ζ-potential (D) of PB100, PB250 and PB800 in HEPES buffer (10 mM, pH7.4) using ZetaSizer Nano ZS (Malvern Instruments, Ltd.).

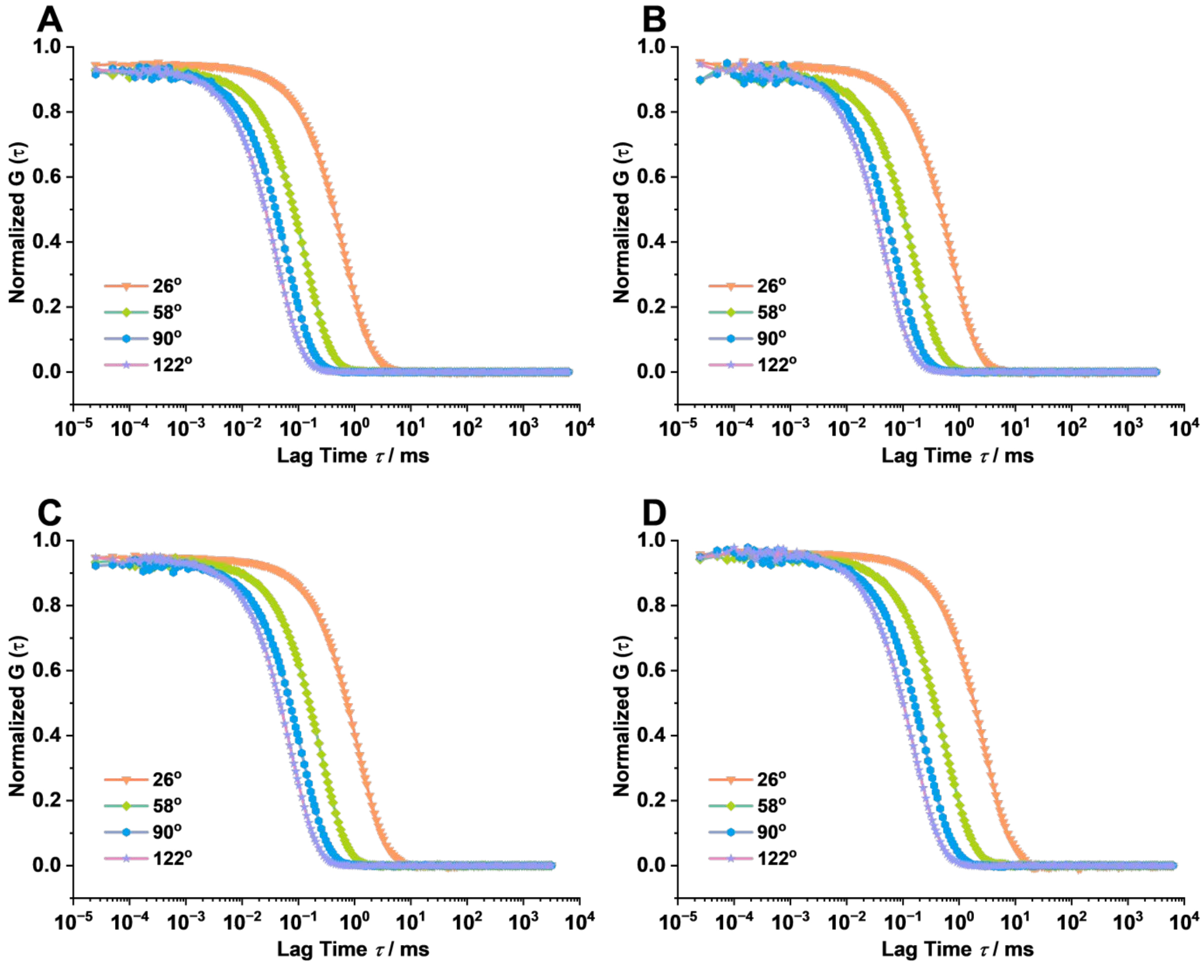

Figure S19. Multi-angle DLS measurements of peptobrushes in PBS. Representative autocorrelation function of PB50 (A), PB100 (B), PB250 (C) and PB800 (D) given for a representative measurement at angle of 26° (orange), 58° (green), 90° (blue), and 122° (purple).

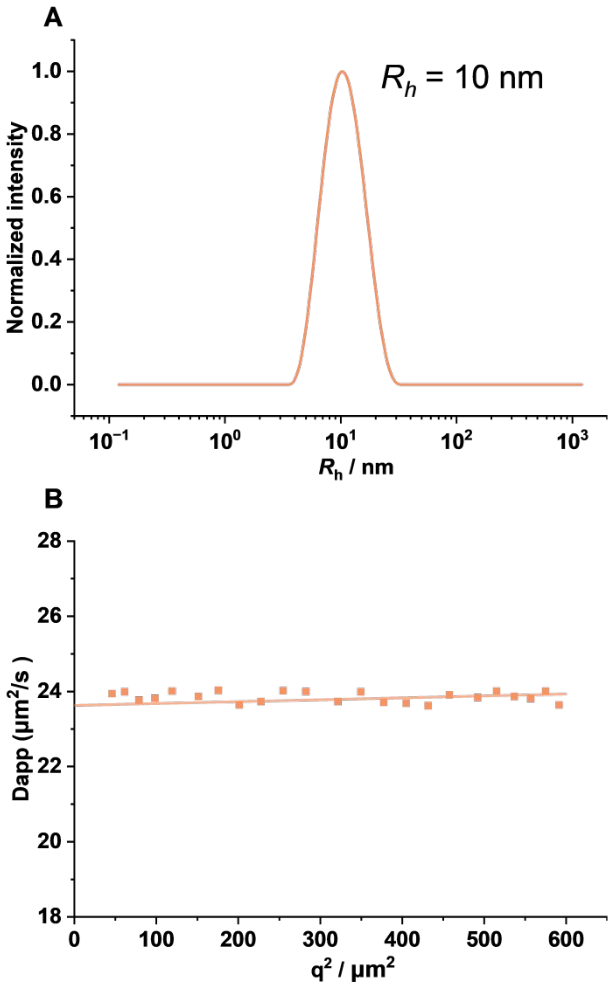

Figure S20. Representative size distribution at 90° (A) and multi-angle DLS analysis (B) of PB50.

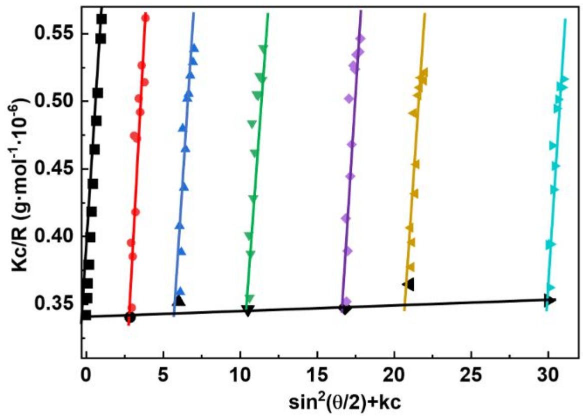

Figure S21. Zimm plot obtained from SLS measurements of PB800 in PBS.

Representative SFM analysis:

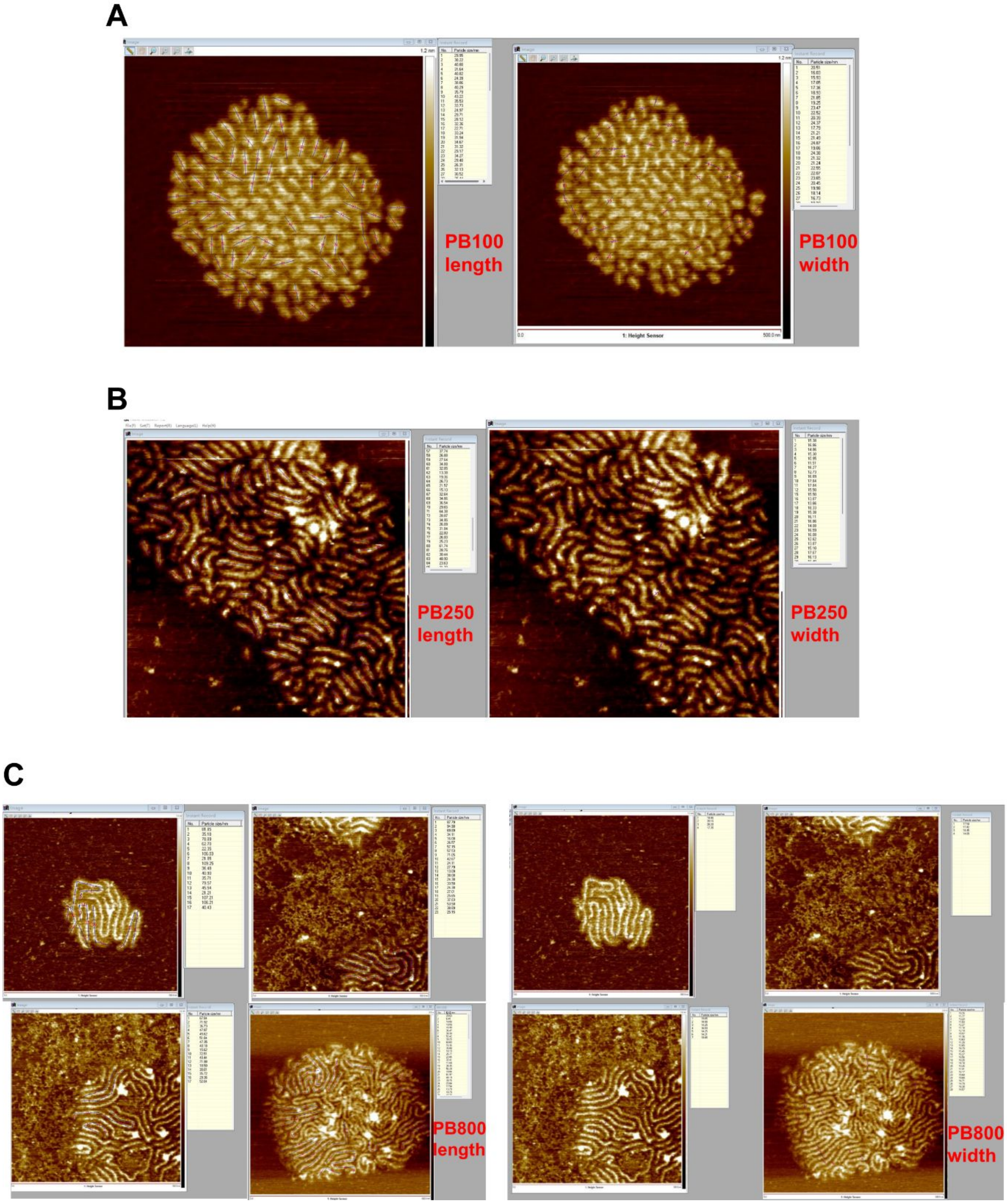

Figure S22. SFM length and width analysis of PB100 (A), PB250 (B), and PB800 (C) by NanoMeasure 1.2.

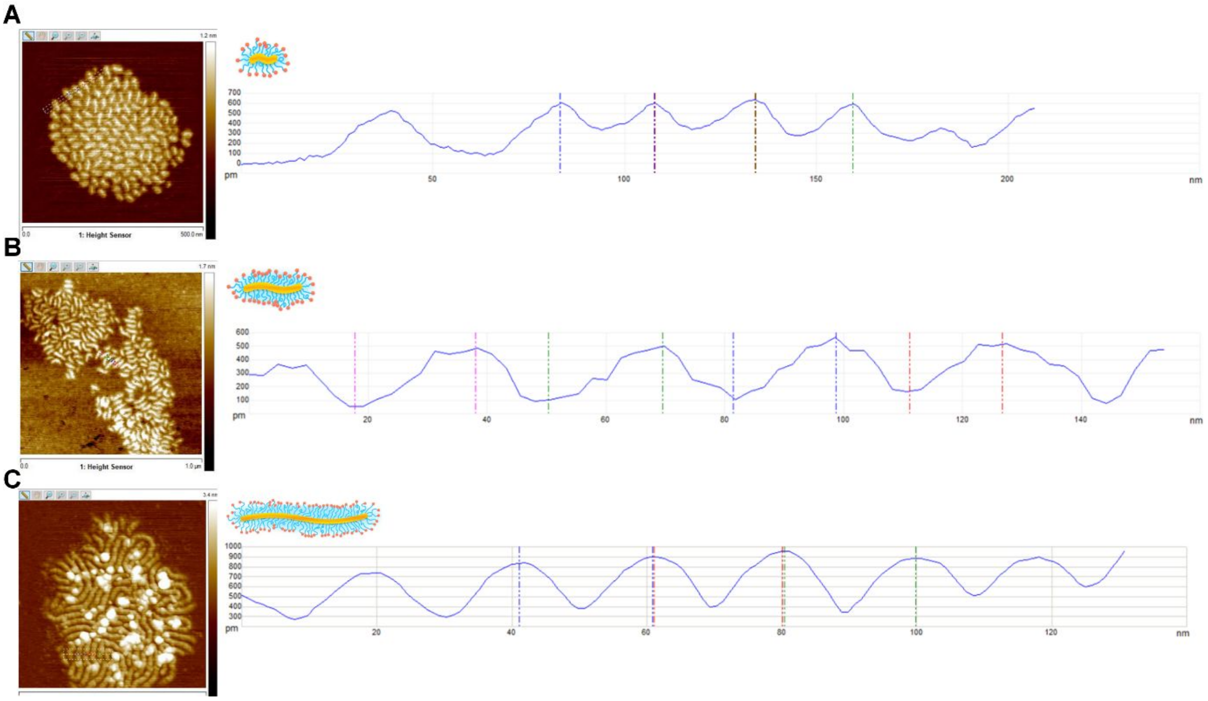

Figure S23. Average SFM height analysis of PB100 (A), PB250 (B), PB800 (C) (n=6).

Representative peptobrush degradation assay analysis:

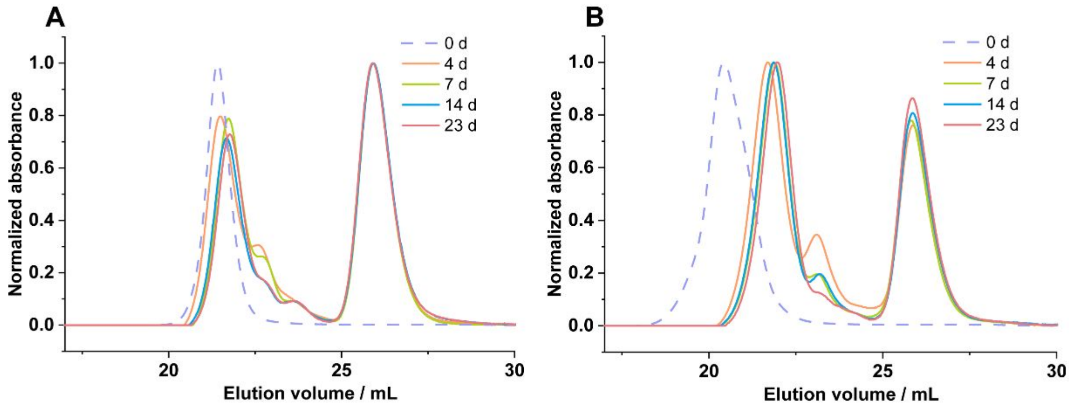

Figure S24. Representative HFIP-SEC curves of PB100 (A) and PB250 (B) at defined time point when being exposed to natural proteases *Streptomyces griseus* at 37 °C ($W_{proteases}/W_{PB}$, 3:2).

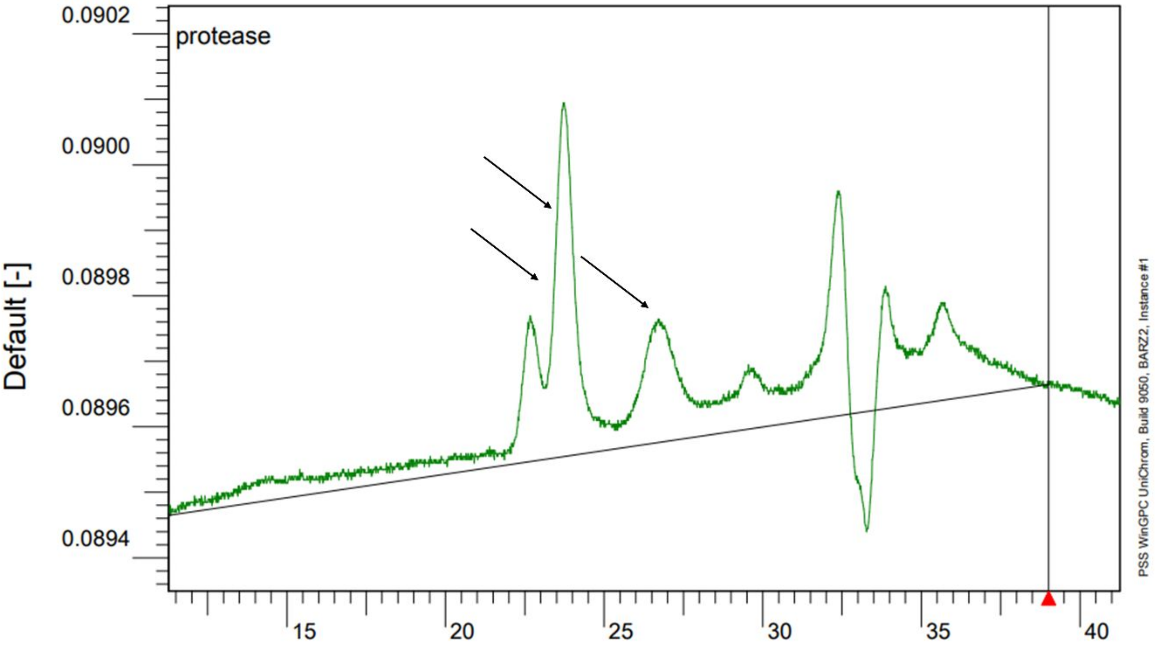

Figure S25. 1 mg·mL^{-1} protease prepared in HFIP and monitored by HFIP-SEC after being filtered by 220-nm filters. No UV-absorption peak was observed due to its poor solubility in HFIP.

Representative MTT assay analysis:

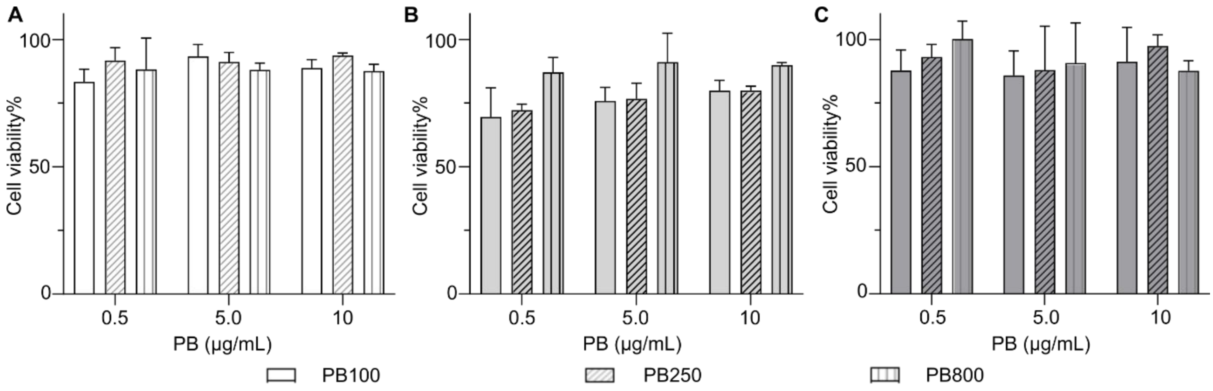

Figure S26. Cell viability (%) of Hela (A), Raw264.7 (B) and Jurkat cells (C) after being exposed to polymer brushes. Peptobrushes in PBS were directly applied onto cells at a final concentration of 0.5, 5.0 and 10 µg mL^{-1}. After 24 hours' exposure, cells were harvested and quantified using a standard MTT assay. All experiments were performed in triplicates (n = 9). Statistical significance was evaluated using two-way ANOVA with multiple comparison correction. A p-value < 0.05 was considered significant.

Additional Information:

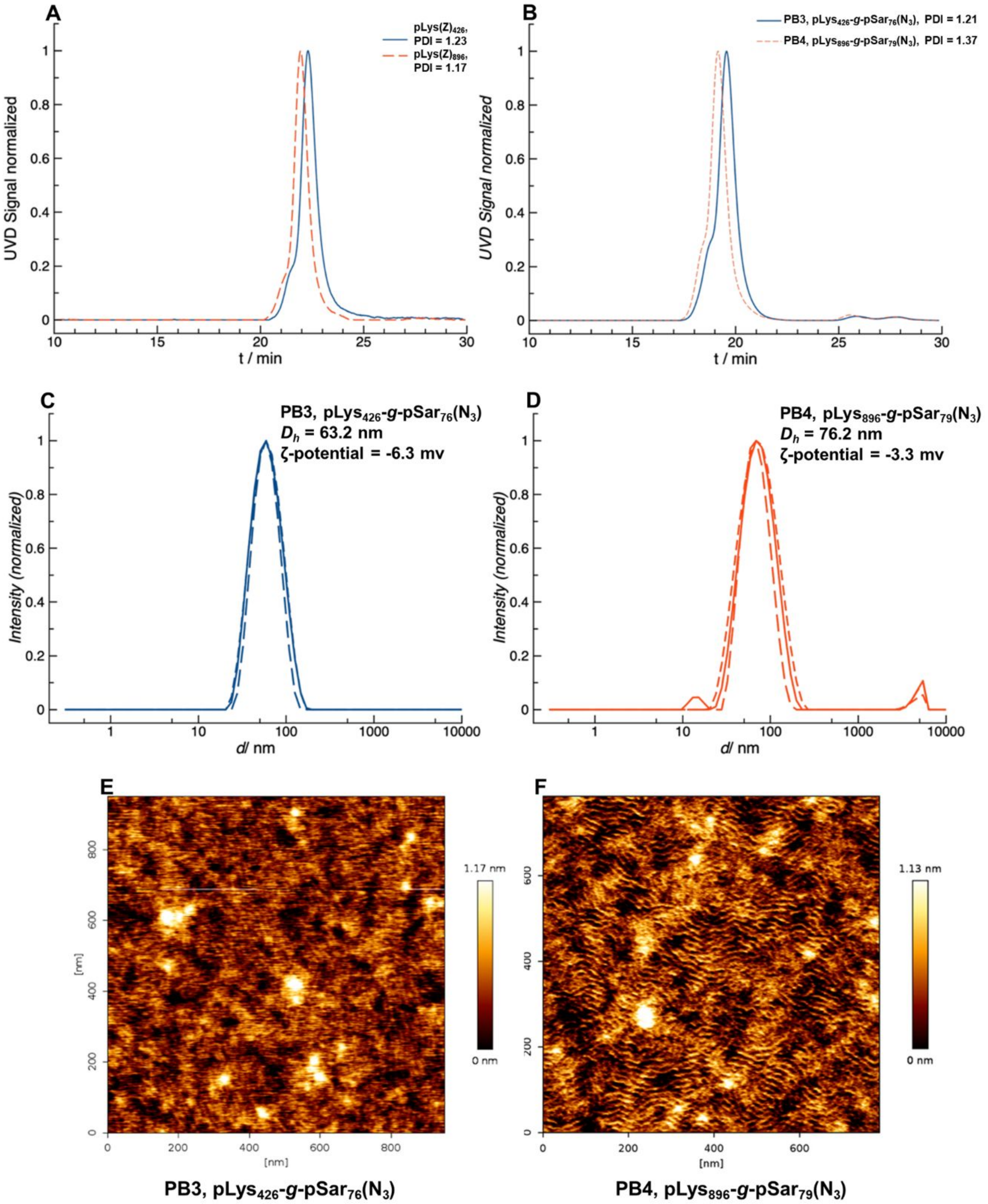

Figure S27. Characterization of house-synthesized PB430 and PB900. Analytical SEC curves of house-synthesized pLys(Z)$_{400}$ and pLys(Z)$_{800}$ backbones (A) and Peptobrushes (B) in HFIP; Hrdyodynamic diameter and ζ-potential was measured by ZetaSizer Nano ZS (C, D); Representative AFM images of PB430 (E) and PB900 (F).
